# Murine Aβ over-production produces diffuse and compact Alzheimer-type amyloid deposits

**DOI:** 10.1186/s40478-015-0252-9

**Published:** 2015-11-14

**Authors:** Guilian Xu, Yong Ran, Susan E. Fromholt, Chunhua Fu, Anthony T. Yachnis, Todd E. Golde, David R. Borchelt

**Affiliations:** Center for Translational Research in Neurodegenerative Disease, University of Florida, Gainesville, FL 32610 USA; McKnight Brain Institute, University of Florida, Gainesville, FL 32610 USA; SantaFe HealthCare Alzheimer’s Disease Research Center, University of Florida, Gainesville, FL 32610 USA; Department of Neuroscience, University of Florida, 1275 Center Dr., Box 100159, Gainesville, FL 32610 USA; Department of Medicine, University of Florida, Gainesville, FL 32610 USA; Department of Pathology, University of Florida College of Medicine, University of Florida, Gainesville, FL 32610 USA

## Abstract

**Introduction:**

Transgenic overexpression of amyloid precursor protein (APP) genes that are either entirely human in sequence or have humanized Aβ sequences can produce Alzheimer-type amyloidosis in mice, provided the transgenes also encode mutations linked to familial Alzheimer’s Disease (FAD). Although transgenic mice have been produced that overexpress wild-type mouse APP, no mice have been generated that express mouse APP with FAD mutations. Here we describe two different versions of such mice that produce amyloid deposits consisting of entirely of mouse Aβ peptides. One line of mice co-expresses mouse APP-Swedish (moAPPswe) with a human presenilin exon-9 deleted variant (PS1dE9) and another line expresses mouse APP-Swedish/Indiana (APPsi) using tetracycline-regulated vectors (tet.moAPPsi).

**Results:**

Both lines of mice that produce mouse Aβ develop amyloid deposits, with the moAPPswe/PS1dE9 micedeveloping extracellular compact, cored, neuritic deposits that primarily localize to white matter tracts andmeningial layers, whereas the tet.moAPPsi mice developed extracellular diffuse cortical/hippocampal deposits distributed throughout the parenchyma.

**Conclusions:**

These findings demonstrate that murine Aβ peptides have the capacity to produce amyloid deposits that are morphologically similar to deposits found in human AD provided the murine APP gene harbors mutations linked to human FAD.

**Electronic supplementary material:**

The online version of this article (doi:10.1186/s40478-015-0252-9) contains supplementary material, which is available to authorized users.

## Introduction

**Fig. 1:**
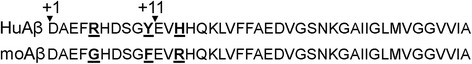
Comparison of human and mouse Aβ sequences. The two primary sites of BACE1 cleavages, designated +1 and +11, are noted by arrowheads above the sequences. The 3 sequence differences between human and mouse Aβ at residues 5, 10 and 13 are underlined in bold

Mutations in the amino acid sequence of the amyloid precursor protein (APP) influence its cleavage by three types of endo-proteases termed α, β, and γ-secretase [8, 16]. Studies of APP processing by the enzyme BACE1 have demonstrated that this enzyme, which is responsible for the cleavage event at the amino terminus of Aβ, can cleave APP to produce two possible major amino terminal sites, termed +1 and +11 (Fig. 1). For wild-type APP sequence, BACE 1 generally cleaves APP at the +11 site, and after cleavage by γ-secretase, Aβ peptides 11–40 and 11–42 are produced [6]. A third enzyme, termed α-secretase, cleaves APP within the Aβ peptide sequence to produce peptides such as Aβ17-40 and 17–42 (for review see [8, 16]). Importantly, Aβ11-40/42 and 17–40/42 peptides are generally not found as significant components of amyloid plaque lesions that characterize human AD [13, 28]. The major Aβ peptide found in mature neuritic plaques of human AD is Aβ1-42 [19, 30]. Mutations in APP that cause early-onset AD can influence cleavage of APP by either BACE1, γ-secretase, or α-secretase so that a greater percentage of Aβ1-42 peptides are generated by these processes [for review see [8, 16]. Thus, the early-onset of AD in patients harboring mutations in APP is thought to be due to the accelerated deposition of amyloid, which initiates a cascade of pathogenic events to produce full spectrum of AD symptoms [17].

In the much more common occurrence of sporadic AD, which is pathologically characterized by the presence of amyloid deposits and tau pathology, mutations in APP are not found and instead it has been suggested that some aspect of aging changes the dynamics of Aβ production or clearance to initiate disease. Amyloid deposits similar to human AD have been described in many species of aged animals including non-human primates, dogs, and bears [39]. However, aged rodents (mouse or rat) do not spontaneously develop amyloid deposits. The human and murine amyloid precursor proteins (APP) differ at three amino acid residues within the Aβ peptide sequence (5, 10, and 13) (Fig. 1). These differences have been reported to modulate the binding of metal ions [18], which can influence fibrillogenesis of Aβ peptides *in vitro* [1]. Additionally, cleavage of mouse APP by mouse BACE1 largely produces Aβ11-40/42 instead of the 1–40/42 peptides [6]. These attributes of the murine Aβ peptide sequence appear to diminish the potential for aged mice to spontaneously develop amyloid deposits.

The capacity for murine Aβ to produce amyloid deposits appears to be limited even in aged mice. There have been reports of intracellular accumulations of granular Aβ immunoreactivity in the senescence accelerated strains of mice (SAMP8) (reviewed in [7]) and one study of these mice described plaque-like structures [33]. However, mice that model aspects of Down’s syndrome, including triplication of the mouse APP gene, do not develop AD-like amyloidosis [35]. Moreover, mice that overexpress human presenilin with mutations linked to FAD do not develop amyloid deposits [3, 45] nor do mice with FAD knock-in mutations in murine presenilin [15, 49]. Mice expressing 5-fold excess of wild-type mouse APP also fail to produce amyloid deposits when mated to mice expressing human PS1dE9 despite raising the levels of MoAβ42 by more than 2 fold [21]. Thus, the capacity of mouse Aβ to produce AD-like amyloid pathology was thought to be limited.

Based on these foregoing observations, we hypothesized that sequence variations within the mouse Aβ peptide, as compared to human, may render the mouse peptide unable to form amyloid *in vivo*. However, because of the complex influences of sequences within Aβ on cleavage by α-, β-, and γ-secretase, it is difficult to pinpoint the basis for the limited capacity of mouse Aβ to deposit. To facilitate more direct comparisons between mouse and human Aβ peptides, we generated two strains of transgenic mice that express a mouse APP695 transgene with mutations linked to FAD, with one of these strains also co-expressing human PS1dE9. Both strains of mice produced amyloid deposits with morphologies that resemble human amyloid. Interestingly, however, the distribution and texture of the amyloid deposited in these two strains differs from what we have seen in mice depositing human Aβ. Moreover, the distribution and texture of the amyloid was different depending upon whether human PS1dE9 was co-expressed. These data are consistent with the notion that sequence differences in deposited Aβ peptides and cleavage patterns by γ-secretase may produce distinct types of amyloid pathology.

## Materials and Methods

### Generation of transgenic mice

We generated two different kinds of transgenic mice overexpressing mouse Aβ. To enable direct comparison to existing lines of humanized Aβ mice (e.g. MoHuAPPswe/PS1dE9-Line 85 [24]), we produced mice co-expressing mouse APP695 harboring the Swedish (K595M/N596L) mutation and human PS1dE9 by co-injecting the two transgenes, each driven by its own prion promoter element [20], into fertilized embryos from crosses of C3H/HeJ and C57BL/6J mice (C3/B6 F1). The two transgenes co-integrated and co-segregate as a single locus. This new line of moAPP695swe/PS1dE9 mice was designated D-943.

The other mouse APP transgenic mouse line we produced is similar to previously described inducible MoHuAPPswe/ind (MoHuAPPsi) transgenic mice (line 107 x tTA) [23], except the Swedish (KM/NL) and Indiana (V617F) mutations were incorporated into the mouse APP695 cDNA (moAPPsi). To create this line of mice, we co-injected the tet.PrP.moAPPsi construct with a construct to express GFP in the skin (K14-GFP) to facilitate genotyping [42, 43]. This line of mice was generated in the FVB/NJ strain (to enable visualization of the GFP). To induce transgene expression, the moAPPsi-GFP mice were crossed to CamKII-tTA mice [29] (congenic on the B6 background) to produce trigenic mice that were B6/FVB F1. This combination of strains is identical to that used in a recently published study of tTA/MoHuAPPsi (line 107) mice [31].

This study also describes other lines of mice that have not been previously published. These include a line of mice that expressing a MoHuAPPswe/ind (MoHuAPPsi) transgene under the transcriptional control of the MoPrP.Xho vector. To facilitate genotyping, these mice were created by co-injecting the MoPrP.MoHuAPPsi construct with the K14-GFP construct. These constructs were injected into C3B6 F1 mice and maintained in the same background. The laboratory name for the line of mice identified as expressing the transgene at useful levels was MHSI-695-GFP (Additional file 1: Table S1). Another line of mice described here for the first time is a line created by co-injecting the tetPrP.MoHuAPPPsi construct with the K14-GFP construct to produce mice that could be easily genotyped. The line identified as being the most useful was designated MoHuAPPsi-GFP#2 (Additional file 1: Table S1). To induce transgene expression, this line of mice was crossed to CamKII-tTA mice to produce trigenic mice.

In recognition that transgene names and laboratory line designations can be complicated we have adopted a strategy of simplified naming for the mice used in this study in which the name includes information on the transgene vector, the type of Aβ that is deposited, and any co-injected genes (see Additional file 1: Table S1 and Results).

Other transgenic mice used in this study have been described and fully characterized in earlier publications including mice expressing MoHuAPP695swe (Lines C3.3 and Q2.2) [3, 24, 37]; mice expressing human PS1 harboring the FAD exon-9 deletion (PS1dE9; Line S9) [26]; and mice co-expressing both MoHuAPP695swe and PS1dE9 (Line 85) [24]. All of these mice were maintained on a hybrid background by backcrossing to C3HeJ × C57BL/6J F1 animals obtained from Jackson Laboratories. The generation of the tTA/MoHuAPPsi mice (line 107) has previously been described [23]. In the present study we have used the line 107 mice that have been backcrossed to C57BL/6J mice with B6 congenic CamKII-tTA mice [31]; bigenic male tTA/MoHuAPPsi mice were mated with nontransgenic female FVB/NJ mice to produce experimental animals [31]. All procedures involving animal handling and processing were approved by the University of Florida Institutional Animal Care and Use Committee, following guidelines set forth by the National Institutes of Health.

### Sequential extraction of Aβ and ELISA

When a mouse was harvested, one hemisphere from each mouse brain was frozen on dry-ice and kept at -80 °C, and these tissues were used for ELISA analysis. The extraction and ELISA methods used have been described previously [25], with minor modification. Briefly, the hemi-brain was seperated into forebrain and cerebellum and weighed. According to the brain weight, 6.67 volumes (in μl to mg of brain) of cold Radio-immunoprecipitation Assay Buffer (RIPA buffer; 50mM Tris-HCl, 150mM NaCl, 1 % Triton X-100, 0.5 % Deoxycholate, 0.1 % SDS) with 1x protease Inhibitor Cocktail Tablets Complete (Roche, Indianapolis, IN, USA) was added. The tissue was homogenized by sonication on ice. Equal volumes of homogenate (500μl) were loaded into ultracentrifuge tubes, then centrifuged at 100,000 x g for 1 hour at 4 °C. The supernatants were collected, designated as RIPA soluble Aβ, and stored at -80 °C. This fraction was also used for immunoblot analysis. The pellet was homogenized by sonication in 500 μl of 2 % Sodium Dodecyl Sulfate (SDS) in water with protease inhibitor cocktail. The homogenate was loaded into an ultracentrifuge tube and centrifuged at 100,000 x g for 1 hour at 4 °C. Same as above, the supernatant was collected (SDS soluble) and stored at -80 °C. The pellet was saved for formic acid extraction by sonication in 70 % formic acid followed by centrifugation at 100,000 x g for 1 h at 4 °C. The supernatant (FA soluble) was collected and stored at -80 °C. Before using, the formic acid extracts were neutralized by diluting in 20 volumes of 1M Tris-Base, 0.5M NaH_2_PO_4_. The samples were then assayed by sandwich ELISA with end specific mAbs anti-Aβ42 (1-11-3 Aβx-42 specific, Covance Inc) and mAb 13.1.1 (Aβx-40 specific, Mayo Clinic) for capture and horseradish peroxidase–conjugated mAb 4G8 (Covance Inc.) for detection.

### Immunoblotting for transgene expression

Mice of each genotype were harvested at 2–3 months of age for assessment of gene expression. Both hemi-brains were frozen on dry ice and kept at -80 °C before using. The frozen brains were sequentially extracted by RIPA, SDS and formic acid as described above. Ten microliters (10μl) of RIPA extracted homogenate (equal to ~50 μg of total protein) were loaded onto 4–20 % Tris-Glycine gel for SDS-PAGE. The following primary antibodies were used: rabbit anti-PS1NT antibody against PS1 (1:5000, kind gift of Dr. Gopal Thinakaran, University of Chicago, Chicago, IL, USA), 6E10 (mouse anti-human Aβ monoclonal, 1:1500, Covance, NJ), 22C11 (mouse anti-human and mouse APP N terminus monoclonal, 1:1000, EMD Millipore, MA) and human and mouse SOD1 antibody (m/hSOD1, rabbit polyclonal, 1:2500) [34]. The ECL images were visualized with a FluorChem E imager (Protein Simple, Santa Clara, CA, USA).

### Histology

The mice used for histology were euthanized by exsanguination and perfusion with phosphate buffered saline (PBS) under isoflurane anesthesia. After the brain was removed, one hemi-brain was frozen on dry-ice for biochemical analysis as described above; the other hemi-brain was immersion fixed in 4 % of paraformaldehyde in PBS at 4 °C for 48 h, then was stored in PBS at 4 °C until paraffin processing for sectioning. Tissue sections of 5 μm or 10 μm thickness were used in histological and immunological staining.

Hirano Silver Stain was done according to standard protocol on 10-μm paraffin-embedded sections by Hirano’s modification of the Bielschowsky method [51]. The slides were scanned by Aperio® XT System (Leica Biosystems, Buffalo Gove, IL, USA). Campbell-Switzer Silver Stain was done according to a detailed protocol provided by Dr. Switzer (NeuroScience Associates, Knoxville, TN; Campbell S, Switzer R, Martin T (1987) Alzheimer’s plaques and tangles: a controlled and enhanced silver staining method. Soc Neurosci Abst 13:678) [46]. Thioflavine S Staining followed the Guntern modification of standard protocols [14]. Congo red staining was performed according to standard techniques [24] with the following modification. Deparaffinized slides with 5 μm brain sections were counterstained in hematoxylin, then immersed in Congo red solution (0.5 % Congo red, 2 % NaCl in 80 % ethanol) for 10 min, rinsed and then dehydrated and coverslipped. Slides were examined under polarizing illumination for evidence of amyloid deposition and images were captured with an Olympus BX40 microscope.

Immunohistochemistry and immunofluorescence staining followed standard protocols. Slides were deparaffinized by oven heating followed by immersion in xylene. After rehydration through graded alcohols into water, the slides were steamed in citrate buffer for 30 min [2]. Nonspecific staining was blocked for 1 h with 3 % normal goat serum and 0.1 % Triton-X 100 in PBS. Slides were then placed into primary antibody diluted in PBS with 2 % normal goat serum and incubated overnight at room temperature. Endogenous peroxidase activity was quenched by incubation with 0.3 % hydrogen peroxide in PBS for 10–20 min. After the excess primary antibody was washed away with several changes of PBS, slides were incubated with either the biotin-labeled secondary antibodies followed by diaminobenzidene (DAB) visualization according to the protocols provided by the supplier. The primary antibodies used are: 4G8 anti-mouse or human Aβ monoclonal antibody (1:250, Covance, NJ); 6E10 anti-human Aβ monoclonal antibody (1:250, Covance, NJ); rabbit monoclonal Aβ42 antibody against both mouse and human Aβ 42 (1:500, Invitrogen); mouse anti-GFAP antibody (1: 500, EMD Millipore, MA); rabbit anti-ubiquitin antibody (1:1000, DAKO, CA). The biotin-labeled secondary antibodies used were goat anti-mouse or rabbit IgG (1:500, Vector Laboratories).

### Image processing

The silver stained sections were scanned using the Scanscope XT image scanner (Aperio, Vista, CA, USA) and analyzed using the ImageScope program. Amyloid plaques were digital extracted with the colocalization V9 algorithm (Aperio). Images of the hippocampus, or whole sagittal sections, were digitally cropped to generate the final images.

### Analysis of APP processing in cultured cells

Cell culture and transfection - Mouse neuroblastoma N2a cells (ATCC, CCL-131) were cultured in 5 ml medium containing DMEM/Opti-MEM with 5 % FBS in 60mm dishes at 5 % CO_2_. At 90 % confluence, 4 μg plasmid DNA (moAPPswe, moAPP-WT, MoHuAPPswe, and MoHuAPP-WT, all in pEF-BOS expression vector [32]) was transfected with 8 μl of lipofectamine 2000 according to the manufacturer’s protocol. After 24 h, the original medium was removed and 4ml fresh medium was added. After another 24 h, the medium was collected for Aβ assay.

Immunoprecipitation and mass spectrometry – 50 μl of magnetic sheep-anti-mouse IgG beads (Invitrogen, Grand Island, NY, USA) were incubated with 4.5 μg 4G8 antibody for 30 min at room temperature with constant shaking. The beads were then washed with PBS and incubated with 5 ml of conditioned medium from transfected N2a cell to which 0.1 % Triton X-100 was added for 30 min. Bound beads were washed sequentially with 0.1 % and 0.05 % octyl glucoside (Sigma-Aldrich, St. Louis, MO) followed by water. Samples were eluted with 10 μl 0.1 % trifluoroacetic acid (Thermo Scientific, Rockford, IL, USA) in water. 2 μl of eluate was mixed with an equal volume of saturated α-cyano-4-hydroxycinnamic acid (CHCA) (Sigma-Aldrich) solution in 60 % acetonitrile, 40 % methanol. 1 μl of sample mixture was loaded on CHCA pretreated MSP 96 target plates (Bruker Daltonics Inc., Billerica, MA, USA). The samples were analyzed with a Bruker Microflex (Bruker Daltonics Inc., Billerica, MA, USA) mass spectrometer.

## Results

**Fig. 2:**
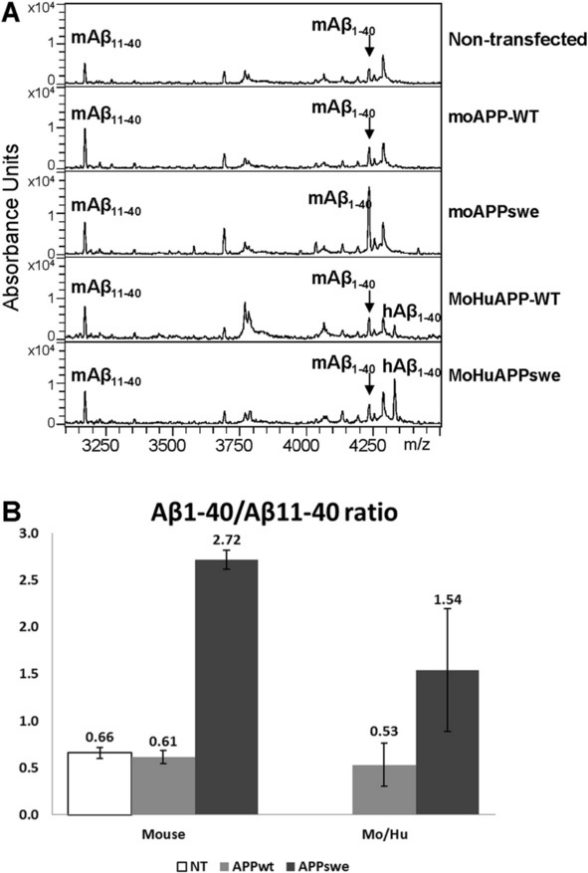
Analysis of Aβ peptides produced by mouse N2a cells transiently expressing mouse and MoHu chimeric APP genes. **a** Examples of mass spectrometry data from the analysis of cell culture medium of mouse N2a cells transiently transfected with expression plasmids encoding moAPP-WT, moAPPswe, MoHuAPP-WT, and MoHuAPPswe. The Aβ in the medium was immunoprecipitated with the monoclonal antibody 4G8 and then analyzed by mass spectrometry. The positions of mouse and human Aβ40 (mAβ40 and hAβ40) are noted on the spectrum traces. **b** Analysis of data from repeated experiments (*n* = 3) demonstrated that the Swedish mutations in mouse APP shift cleavage to produce a greater amount of Aβ1-40 over Aβ11-40. Standard deviation (SD) was shown at the error bars

Prior studies of APP processing in murine cells have demonstrated that murine BACE1 preferentially cleaves wild-type mouse APP at the +11 residue of Aβ [6]. In human cells, human WT APP is also preferentially cleaved at residue +11, but the Swedish double mutations in APP shift cleavage to the +1 residue [6]. To determine whether introducing the Swedish mutations into murine APP produces the same effects, we transfected mouse N2a cells with expression vectors for moAPP695, with and without the Swedish mutation, and analyzed the Aβx-40 peptides produced by immunoprecipitation and mass spectrometry (Fig. 2). For comparison we also expressed WT and Swedish variants of the chimeric MoHuAPP695 cDNAs used previously to produce transgenic mice [3]. Both the moAPP695 and MoHuAPP695 proteins with WT sequences produced primarily Aβ11-40 (Fig. 2a and b), whereas both of these proteins encoding the Swedish mutation [K595N,M596L] produced primarily Aβ1-40 (Fig. 2a and b). Aβ42 levels were too low to quantify accurately in this system, but there was trend of Aβ42 increasing in the N2a cells expressing APP with Swedish mutation.

Satisfied that murine APP with the Swedish mutations would enhance the production of Aβ1-40/42, we proceeded to generate transgenic mice expressing mutant murine APP695. Two constructs were generated; one construct was moAPP695 with the Swedish mutations (moAPPswe) and the other was moAPP695 with the Swedish and Indiana mutations (moAPPsi). These new lines of mice were compared to existing lines of mice in our colony that express a humanized version of moAPP695 (MoHuAPP), in which the Aβ sequence had been modified to produce human Aβ [3]. These original humanized APP mice were generated in this manner to potentially avoid any effects that could arise from any human APP specific activities of holo APP or shed APP ectodomains. Thus, here we are essentially comparing mice that over-express different variations of murine APP that differ by AD mutation encoded and by sequence of the Aβ peptides that would be generated.

To facilitate comparisons of any mice generated with these constructs with existing lines of mice expressing humanized APP constructs, we used two strategies to produce transgenic mice. The moAPPswe constructs were inserted in the MoPrP.Xho vector and co-injected with MoPrP.Xho vectors encoding human PS1dE9 to mimic previously described MoHuAPPswe/PS1dE9-Line 85 mice [24]. The moAPPsi cDNAs were inserted into tetPrP.Xho vectors to mimic previously described tetPrP.MoHuAPPsi-Line 107 mice [23]. Founders for each of the transgene injections were produced and, as described below, we identified one line of mice for each that expressed total APP levels that were comparable to existing lines of MoHuAPP mice. Because of the complicated nomenclature that arises in generating identifiers for different lines of mice, for this paper we identify the mice with short-hand abbreviations for vector used (PrP or tet), sequence of Aβ produced (mo or Hu), and co-expressed transgenes (see below) (Additional file 1: Table S1).

### Analysis of moAPPswe and moAPPsi expression in transgenic mice

**Fig. 3:**
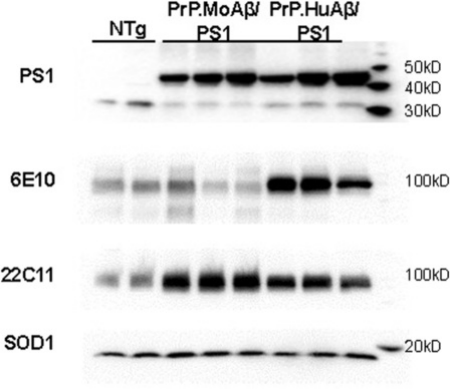
Comparison of APP and PS1 expression levels in PrP.MoAβ/PS1 and PrP.HuAβ/PS1 mice. The brains of 2 non-transgenic (age 1.6–2.4 months) and 3 each PrP.MoAβ/PS1 (Line D-943; aged 2.5–3.3 months) and PrP.HuAβ/PS1 (Line 85, aged 1.6–2.4 months) mice were homogenized in RIPA buffer, the supernatants were analyzed by immunoblotting 4–20% SDS-PAGE gels with antibodies to PS1 (1:5000, top row), human APP (mAb 6E10, 1:1500, second row), total APP (mAb 22C11, 1:1000, third row), or mouse SOD1 (1:2500, fourth row). The mouse SOD1 immunoblot served as a loading control. The levels of PS1dE9 and total APP were similar in the two lines of mice. Each lane contains 50 μg of total protein

A line of moAPPswe/PS1dE9 mice designated as line D-943 (which we identify from here on as PrP.MoAβ/PS1) (Additional file 1: Table S1) was found to express total APP (transgene plus endogenous) at levels comparable to the previously described MoHuAPPswe/PS1dE9-Line 85 mice [24] (from here on identified as PrP.HuAβ/PS1) (Fig. 3). Brain homogenates of 3 different PrP.MoAβ/PS1 mice were compared to brain homogenates of 3 different PrP.HuAβ/PS1 mice by immunoblot with antibodies to human presenilin, the monoclonal antibody 6E10 (prefers human APP/Aβ), the monoclonal antibody 22C11 (recognizes an N-terminal epitope that is shared by all APP constructs used), and SOD1 (a loading control). The immunoblots probed with 22C11 demonstrated similar levels of total APP in the PrP.MoAβ/PS1 and PrP.HuAβ/PS1 mice (Fig. 3). Similarly, the levels of human PS1dE9 protein were similar in the two lines of mice (Fig. 3). The APP expressed in PrP.HuAβ/PS1 mice was much more reactive to the monoclonal antibody 6E10 as expected. Immunoblots probed with the SOD1 antibody demonstrated equal loading (Fig. 3). These data demonstrate that our newly developed PrP.MoAβ/PS1 mice express the transgene at levels that are similar to our previously characterized PrP.HuAβ/PS1 mice [24].

In producing the tetPrP.moAPPsi mice, we employed a strategy in which we co-injected the APP transgene with a transgene construct that encodes green fluorescent protein (GFP) under the transcriptional control of the K14 promoter (see Methods). We have recently used this strategy in producing a series of transgenic mice that express N-terminal fragments of mutant huntingtin [42, 43]. Expression of the GFP in skin provides an easy marker to identify transgenic animals and lowers the cost of maintaining the lines of mice. To induce expression, the tetPrP.moAPPsi mice were crossed to mice expressing the tetracycline-transactivator construct under the transcriptional control of the CamKII promoter in a strategy identical to what was previously used to express MoHuAPPsi [23]. The levels of total APP (detected with monoclonal antibody 22C11) in bigenic tTA/moAPPsi [hereafter identified as tet.MoAβ(GFP)] mice (Additional file 1: Figure S1, lane 5 and 6) were similar to that of the PrP.MoAβ/PS1 and PrP.HuAβ/PS1 mice, but much lower than two different lines of tet.HuAβ mce (bigenic for CamKII-tTA and tet.MoHuAPPsi; see Additional file 1: Table S1) that express humanized APP that we currently have in our colony. One of these lines of tet.HuAβ mice, designated line 107, has previously been described [23] [hereafter designated tet.HuAβ(107)], the other line, carrying the laboratory name tetMoHuAPPsi-GFP#2 [hereafter identified as tet.HuAβ(GFP)], has not been described previously and is a new line of mice that was made by co-injection of the K14-GFP vector. In our colony, there is also a line of mice that expresses MoHuAPPsi via the constitutive MoPrP.Xho vector (also co-injected with K14-GFP, carrying the laboratory name of MHSI-695-GFP) that has not been previously described [hereafter designated PrP.HuAβ(GFP)]. Fortunately, the levels of total APP in the brains of bigenic tet.MoAβ mice were similar to the levels in PrP.HuAβ(GFP) mice (Additional file 1: Figure S1), providing a line of mice that could be directly compared to the tet.MoAβ mice. Collectively, these lines of mice provide a basis for comparison of amyloid pathology in mice that produce mouse or human Aβ while controlling for the effects of co-expressed human PS1dE9 and the co-injection of K14-GFP vectors to mark transgenic animals.

### Analysis of amyloid deposition in the PrP.MoAβ/PS1 and PrP.HuAβ/PS1 mice

**Fig. 4:**
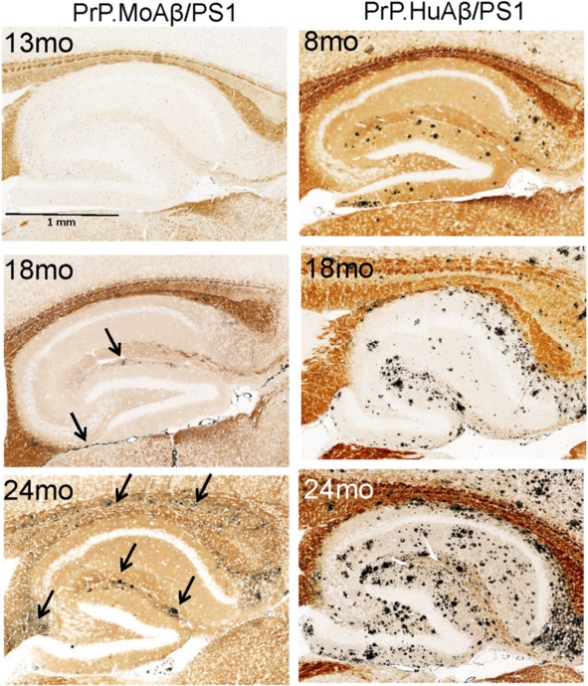
Distribution of amyloid deposition in PrP.MoAβ/PS1 and PrP.HuAβ/PS1 mice. Representative images of the hippocampus from tissue sections stained by Campbell-Switzer silver stain are shown. The approximate age of the animal at the time of sacrifice is shown. The amyloid appears as the dark black material. The images shown are representative of images from an analysis of at least 3 animals at each age and each genotype with at least 6 individual tissue sections taken from similar levels for each animal

Our first observation was that the age at which amyloid deposits first appear in the PrP.MoAβ/PS1 mice was much later than that of the PrP.HuAβ/PS1 mice. Amyloid deposits first appear in the hippocampus of PrP.HuAβ/PS1 mice at about 6 months of age and are readily visible in silver stains by 8 months of age (Fig. 4). Amyloid deposits were first visible in the hippocampus of PrP.MoAβ/PS1 mice at 13 months old (1 out of 4 observed), but then became consistently visible in 18 month old animals. The frequency of deposits was slightly higher in 24 month old mice, visible in the corpus callosum and the area adjacent to the hippocampal fissure (Fig. 4, arrows). Still, the amyloid burden in the hippocampus of 24 month old PrP.MoAβ/PS1 mice was considerably lower than that of 8 month old PrP.HuAβ/PS1 mice (Fig. 4).

In the brains of 24 month old PrP.MoAβ/PS1 mice we observed amyloid deposits concentrated in the subpial layers of the cortex and cerebellum, and the white matter tracts of the corpus callosum (Additional file 1: Figure S2 and S3). By contrast, as previously reported [24], the plaques in the PrP.HuAβ/PS1 mice were distributed throughout the cortex and hippocampus. In the cerebellum of PrP.HuAβ/PS1, the amyloid distribution was more similar to that of the PrP.MoAβ/PS1 mice, being concentrated in subpial layers (Additional file 1: Figure S2 and S3). Thus, in the PrP.MoAβ/PS1 mice, both the age of onset and the pattern of deposition of amyloid differed from that seen in PrP.HuAβ/PS1 mice.

**Fig. 5:**
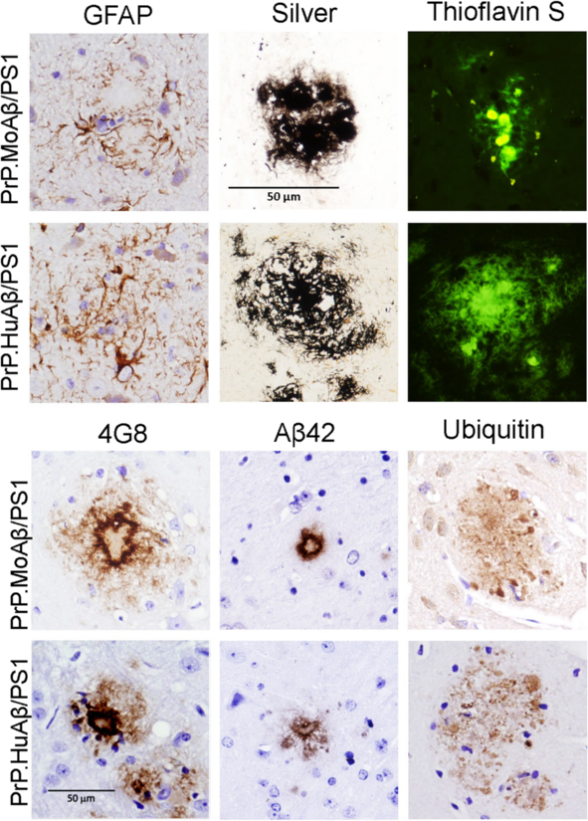
Histologic comparison of amyloid plaque morphology in PrP.MoAβ/PS1 and PrP.HuAβ/PS1 mice. Representative images of cortical amyloid plaques in tissue sections stained with antibodies to GFAP, Aβ (mAb 4G8 and rAb Aβ42) and ubiquitin, and by Campbell-Switzer silver stain or Thioflavin-S staining. The images shown are representative of an analysis of at least 3 animals at each age and each genotype with at least 6 individual tissue sections taken from similar levels for each animal. All the images shown were obtained at the same magnification (40x) and digitally cropped

At the microscopic level, the morphology of amyloid deposits in the PrP.MoAβ/PS1 and PrP.HuAβ/PS1 mice were similar (Fig. 5): both showed GFAP immunoreactive astrocytes in close proximity to the plaques, both displayed a compact morphology, both stain with thioflavin S (Fig. 5), and show congo red birefringence and fluorescence (Additional file 1: Figure S4). Immunostaining with antibodies to Aβ (monoclonal Ab 4G8 and Aβ42 specific) demonstrated that dense cored plaques in the PrP.MoAβ/PS1 were similar to deposits in PrP.HuAβ/PS1 mice (Fig. 5). There was no remarkable staining with Aβ42 specific antibodies within cell bodies in either of these lines of mice (not shown). Similarly, immunostaining with ubiquitin revealed neuritic pathology in close proximity to the deposits in PrP.MoAβ/PS1 mice (Fig. 5). Similar to what we have previously described for the PrP.HuAβ/PS1 mice [50], there was no evidence of tau neuritic pathology or obvious neuronal loss in hippocampal structures in the PrP.MoAβ/PS1 mice (data not shown).

### Comparison of amyloid deposition in tet.MoAβ(GFP), tet.HuAβ(107), and tet.HuAβ(GFP) mice

**Fig. 6:**
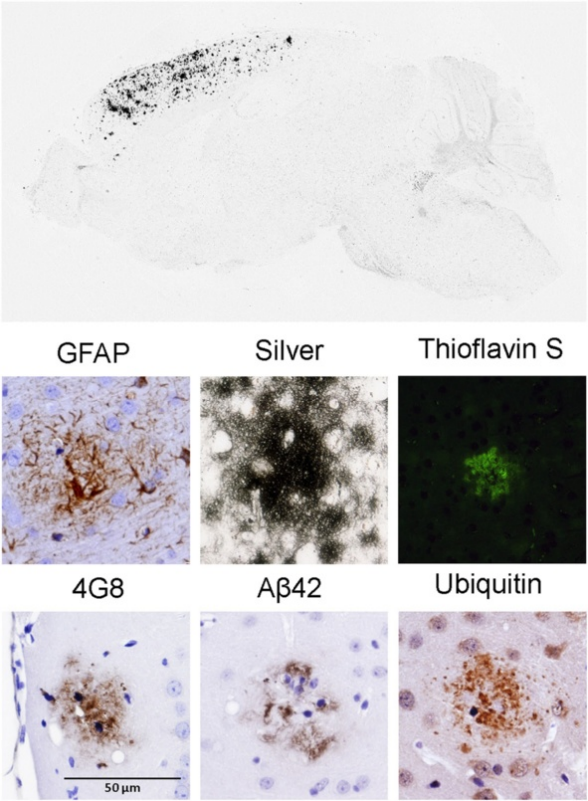
Analysis of amyloid distribution and morphology in tet.MoAβ(GFP) mice. The overall distribution of deposited amyloid is shown by digital extraction of staining by Cambell-Switzer silver staining. Additional representative images of cortical amyloid in tissue sections stained with antibodies to GFAP, Aβ (mAb 4G8 and rAb Aβ42) and ubiquitin, and by Campbell-Switzer silver stain or Thioflavin-S staining. The images shown are representative of images from an analysis of at least 3 animals at each age and each genotype with at least 6 individual tissue sections taken from similar levels for each animal. The example of Thioflavin-S staining of the tet.MoAβ(GFP) tissue represents the maximal fluorescent intensity that was observed. All the images were shown at the same amplification after cropping from 40x microscope images

The distribution of the amyloid deposits in the tet.MoAβ(GFP) contrasted starkly to that of the PrP.MoAβ/PS1 mice. The tet.MoAβ(GFP) mice developed deposits primarily throughout the parenchyma of the rostral cortex with lesser amounts of amyloid in the hippocampus (Fig. 6 and Additional file 1: Figure S5). Because the CamKII promoter drives expression of tTA and the responsive tet.PrP-moAPPsi transgene, primarily in the forebrain [23], the absence of amyloid pathology in the cerebellum is expected in these mice. Somewhat surprisingly, the level of amyloid in hippocampus in 15–18 month old tet.MoAβ(GFP) mice was variable (Fig. 6; undetectable in the example shown), but by 24 months of age hippocampal deposits were relatively abundant (Additional file 1: Figure S6). The deposited amyloid in the tet.MoAβ (GFP) mice was much more diffuse; most of these deposits were not stained with Thioflavin-S, and those that did show some staining were only faintly fluorescent (Fig. 6). The amyloid deposited in these mice did not produce birefringence with congo red (data not shown). Still, these diffuse deposits showed GFAP reactive astrocytes in close proximity and evidence of ubiquitin positive neuritic structures (Fig. 6). Overall, the data demonstrate abundant amyloid deposition in the mice, but the amyloid largely appeared to be diffuse deposits with little or no cored deposits.

Given the rather unique appearance of the amyloid pathology in the tet.MoAβ(GFP) mice, we provide here a comparison to two strains of mice that deposit HuAβ, using tet-regulated vectors and CamKII-tTA drivers to expressed humanized APP. One of these lines of mice is a previously described line that designated line 107 [tet.HuAβ(107)] [23]. The other line is a line developed more recently in which the tet-MoHuAPPsi transgene was co-injected with the K14-GFP construct [tet.HuAβ(GFP)], essentially a comparable strategy to what was used to generate the tet.MoAβ(GFP) mice. These two lines of tet.HuAβ mice consistently developed cored neuritic amyloid plaque pathology in both the cortex and hippocampus at about the same time (see Additional file 1: Figure S5). In both of these models, there was little evidence of amyloid deposition around blood vessels supplying the cortical parenchyma. The majority of amyloid that one could potentially describe as vascular was restricted to the subpial layers (Additional file 1: Figure S5). Thus, in two distinct lines of tet.HuAβ mice, the amyloid pathology that developed was quite distinct from that of the tet-MoAβ mice.

To determine how the co-expression of PS1dE9 influenced the morphology and distribution of amyloid, we examined the amyloid pathology in mice that express MoHuAPPsi that were made by co-injection with K14-GFP [PrP.HuAβ(GFP)]. The APP gene expressed in these mice is identical to what is expressed in the tet.HuAβ(107) and tet.HuAβ(GFP) mice. Amyloid pathology found in these PrP.HuAβ(GFP) mice was similar to that of the PrP.HuAβ/PS1 mice (Additional file 1: Figure S7). The only mentionable difference was that the level of diffuse amyloid pathology in the PrP.HuAβ(GFP) mice was higher than that of the PrP.HuAβ/PS1 mice. Additionally, similar to the PrP.MoAβ/PS1 and PrP.HuAβ/PS1mice, the PrP.HuAβ(GFP) mice developed amyloid pathology in the choroid plexus bordering the hippocampus (Additional file 1: Figure S7).

### Analysis of Aβ peptides deposited in the brains of MoHuAPP and moAPP transgenic mice

Analysis of Aβ peptides deposited in the brains of the different transgenic mice by ELISA, with antibodies specific to Aβ40 and 42, produced data that paralleled the neuropathologic analyses and revealed an intriguing difference between the HuAβ and MoAβ depositing mice. As expected from the neuropathological data, the levels of insoluble Aβ increased as the animals aged, with the levels of insoluble Aβ in the brains of the PrP.HuAβ/PS1 mice (both SDS and formic acid extractable) being higher than that of the PrP.MoAβ/PS1 mice (Additional file 1: Figure S8A). Unexpectedly, there was a stark difference in the ratio of insoluble Aβ40 to Aβ42 in the brains of these mice. In the PrP.HuAβ/PS1 mice, the ratio of Aβ42 to 40 ranged from 2:1 to 10:1 (Additional file 1: Figure S8B), whereas in the forebrains of the PrP.MoAβ/PS1 mice the ratio ranged from 10:1 to 20:1 (Additional file 1: Figure S8B). These data indicate that murine Aβ40 is far less prone to co-aggregate with Aβ42 than the human peptides.

Another striking feature observed in our ELISA analysis was that almost all of the Aβ in the brains of the tet.MoAβ(GFP) mice was soluble in SDS with very low levels in the formic acid fraction (Additional file 1: Figure S9); data that are consistent with the diffuse morphology of the amyloid deposits in this line of mice. The ratio of Aβ42 to 40 in the SDS-soluble fraction skyrocketed as the mice aged (Additional file 1: Figure S9), indicating that the vast majority of the deposited peptide was Aβ42. Thus, in both of our strains of mice that developed mouse Aβ amyloid, the primary deposited peptide was Aβ42.

### Cerebellar amyloid

Cerebellar amyloid deposits in the PrP.HuAβ/PS1 mice were observed when these mice were first analyzed more than 10 years ago [23], but the significance of such pathology was enigmatic and thus not fully described. The first mice that co-expressed humanized APP (MoHuAPPswe) and PS1dE9 mice were produced by crossing mice that independently expressed the genes, and in a re-examination of these animals we document that the frequency of cerebellar amyloid deposits was very low (Table 1). A similarly low frequency of cerebellar amyloid was observed with a second line of MoHuAPPswe mice (line Q2-2) that was crossed to the PS1dE9 mice (Table 1). Here, we document the features of cerebellar amyloid in the PrP.HuAβ/PS1 and the PrP.MoAβ/PS1 mice made by co-injecting the transgenes.

**Table 1:** Comparison of the frequency of cerebellar amyloid deposits among the different lines of transgenic mice analyzed

Aβ	Human					Mouse
Transgenes	PrP.HuAβ/PS1				PrP.HuAβ	PrP.MoAβ/PS1
Line	85	57	C3.3xS9	Q2.2xS9	MHSI-695	D-943
Age (mo)*	16	21	18	22	20	24
Hippocampus	+++	+++	+++	+++	+++	++
Cerebellum	+++	+++	+	-	**+**	++

+: 1–5 plaques, ++: 6–20 plaques, +++: >20 plaques

*Age analyzed

**Fig. 7:**
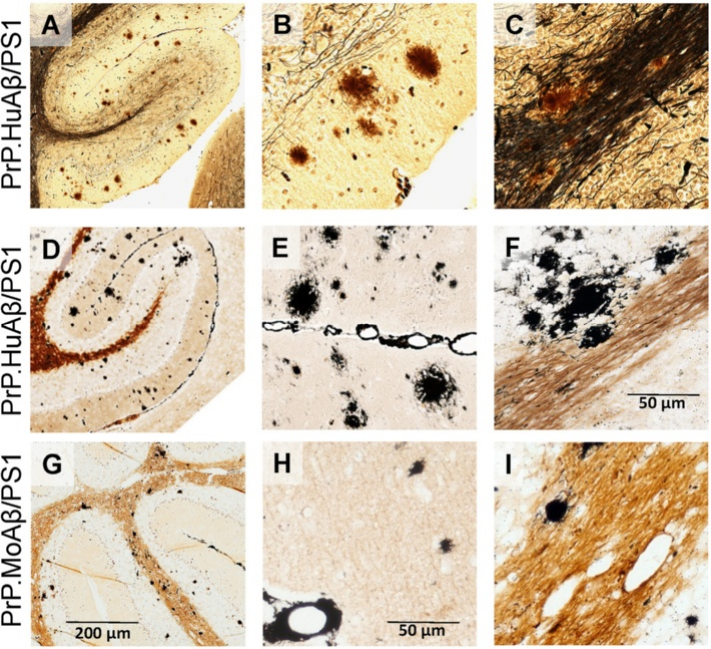
The morphology of amyloid plaques in the cerebellum of D-943 and Line 85 mice. **a**–**c** Representative images of cerebellum stained by Hirano silver staining. **d**–**i** Representative images stained by Campbell-Switzer silver staining. **a**–**f** PrP.HuAβ/PS1 mice (line 85); **g**–**i** PrP.MoAβ/PS1 mice. **a**, **d** and **g** 10x objective; **b**, **c**, **e**, **f**, **h** and **i**) 40x objective. **b**, **e** and **h**) Cerebellar grey matter plaques; **c**, **f** and **i**) Cerebellar white matter plaques

Cerebellar amyloid occurs in two distinct lines of PrP.HuAβ/PS1 mice; the widely-used Line 85 mice and a sister line mice designated Line 57 [20] (Table 1). Although the amyloid plaques in the cerebellum of the PrP.HuAβ/PS1 (Line 85) mice were generally small, Hirano and Campbell-Switzer silver stains revealed deposits that were morphologically similar to deposits observed in forebrain structures (Fig. 7). For example, amyloid deposits in both the grey and white matter of the cerebellum of these mice appeared as compact deposits (Fig. 7a–f). Similar deposits were observed in the PrP.MoAβ/PS1 mice (Fig. 7), but here the deposits were small, very compact structures that were more frequent in the white matter tracts (Fig. 7g–i). Previous studies of patients with the PS1dE9 mutation have described non-neuritic amyloid plaques in the cerebellum [9, 47, 48]. We have previously described the utility of ubiquitin immunostaining as a means to identify neuritic plaques [22], and we used this approach to determine whether the cerebellar amyloid deposits in these mice showed neuritic profile. High power images of deposits in cerebellum reveal eosinophilic cores of amyloid surrounded by ubiquitin immunoreactive neurites (Additional file 1: Figure S10). Analysis of the Aβ peptides deposited in the cerebellum of these mice demonstrated that both Aβ42 and 40 are deposited in the cerebellum of the PrP.HuAβ/PS1 (line 85) mice, ratio ranges from 5:1 to 10:1. In the PrP.MoAβ/PS1 mice the ratio was much higher, all greater than 15:1 (see Additional file 1: Figure S8B). In general, the architecture of cerebellar amyloid plaques in the PrP.HuAβ/PS1 and PrP.MoAβ/PS1 mice was very similar to that of deposits in the hippocampus and cortex, although deposits in the cerebellum of each line were generally smaller than the deposits in the forebrains of the same animal.

## Discussion

Our findings provide the first examples of amyloid pathology formed by rodent Aβ peptides. Unlike other mammalian species, aged rodents have not been reported to develop Aβ deposits unless they express APP genes encoding human Aβ sequences [12, 38] or harbor modified APP genes in which the exon encoding the Aβ domain has been modified to encode human Aβ [10, 36]. The lack of amyloid pathology in aged rodents cannot be fully explained by the inherent abilities of mouse and human Aβ peptides to form fibrillar aggregates since both peptides show similar propensity to aggregate *in vitro* [11]. However, it is now well recognized that the processing of APP by its three secretases is heavily influenced by the sequence of the peptide and adjacent sequences in the APP holoprotein (for review see [8, 16]), with the processing by BACE1 having a major influence on whether Aβ1-40/42 or 11–40/42 is generated [6]. For wild-type moAPP, cleavage by murine BACE favors the production of the non-amyloidogenic Aβ11-40/42 peptides. Thus, whether murine Aβ1-40/42 is really incapable of fibrillogenesis had not rigorously been tested *in vivo*. Our study now provides the rigorous test by introducing moAPP transgenes harboring mutations linked to FAD that shift cleavage of APP to favor the production of moAβ1-40/42. Our findings indicate the moAβ is fully capable of forming both diffuse and compact amyloid plaques *in vivo.* For reasons that we have yet to elucidate, the morphology of the deposited amyloid in these models was heavily influenced by the mode of transgene expression and whether human PS1dE9 was co-expressed. Nevertheless, the data provide the first definitive proof that Aβ peptides of murine amino acid sequence can produce amyloid plaques that are morphologically similar to human amyloid deposits.

The present report also documents, for the first time, the frequency and severity of cerebellar amyloid deposits in the mice that co-express mutant APP and mutant PS1. In the initial iterations of bigenic APP/PS1dE9 mice, generated by crossing 2 independent lines of mice, cerebellar amyloid deposits were inconsistently observed, and present at a low frequency when observed (see Table 1). However, in the more commonly used bigenic APPswe/PS1dE9 mice, generated by co-injection of the transgenes [20, 24], the levels of cerebellar amyloid are considerably higher. The more often used APPswe/PS1dE9 mice Line 85 mice (Jax Strains 005864 and 004462) were created at the same time as a second line, designated line 57. Mice from line 57 were first described by Jankowsky et al. [20], whereas mice from Line 85, which initially bred poorly, were not described until later [24]. For enigmatic reasons, the Line 85 mice emerged as the more used line mice that is deposited in the Jackson Laboratories. We have routinely aged mice from Line 85 out to advanced ages and have never observed obvious gait abnormalities. However, mice of this line have been tested on the rotarod device and have been reported to show deficits [40]. Thus, the presence of amyloid pathology in the cerebellum of the APPswe/PS1dE9 mice may produce moderate deficits in motor performance.

Although cerebellar amyloid pathology is rare in sporadic AD, studies of Finnish patients with the PS1dE9 mutation have documented frequent amyloid deposits in the cerebellum [47]. This unusual pathology correlated to unusual symptoms of paraparesis in this pedigree [9]. In this same pedigree, pathologic descriptions of patients with the PS1dE9 mutation demonstrated unusual amyloid plaque pathology with structures termed cotton wool plaques along with abundant non-cored senile plaques in the cerebral cortex [9, 47]. Thus, the presence of cerebellar amyloid pathology in mice that co-express mutant APP and PS1dE9 may represent a partial reproduction of the human pathology. However, we also observed amyloid pathology in the pia surrounding the cerebellum of PrP.HuAβ(GFP) mice, which do not express PS1dE9. Moreover, McGowen et al observed cerebellar amyloid in mice that express fusion proteins of Bri-Aβ42 via the MoPrP.Xho vector [30]. Thus, there may also be influences of the vector used to produce the transgenic expression on the distribution of amyloid deposition.

Presenilins are integral components of the γ-secretase complex, which is one of two primary enzymes involved in cleaving APP to produce Aβ40 and Aβ42 [for review see [8, 16]. In regard to the generation of Aβ peptide, the major consequence of AD-linked mutations in PS1 and PS2 on APP processing is to shift the processivity of γ-secretase cleavage such that more Aβ42 is produced relative to shorter Aβ peptides such as Aβ38 (for review see [8, 16]). This shift in abundance promotes the deposition of Aβ40 and 42 into senile plaques. Multiple laboratories have demonstrated that the PS1dE9 mutation was among those that produce the most robust increase in the production of Aβ42 [5, 41]. Additionally, when mutant human PS1dE9 is overexpressed, it competes for other cofactors of γ-secretase (nicastrin, Pen2, Aph1) causing a displacement of the endogenous PS1 from this complex [26, 44]. Thus, we can reasonably expect mice that co-express APPswe with PS1dE9 may produce a slightly different spectrum of Aβ peptides than mice in which γ-secretase contains only endogenous mouse PS1. This difference in the spectrum of peptides produced could modulate the location and architecture of deposited amyloid.

Both of our lines of mice depositing MoAβ peptides displayed novel patterns of amyloid deposition and distinct plaque morphologies (Table 2). Notably, regardless of distribution or morphology, all types of deposits were found to include ubiquitin-immunoreactive profiles indicative of neuritic pathology. Collectively, we compare 3 lines of mice that use the MoPrP.Xho vectors to 3 lines that use CamKII-tTA + tetPrP.Xho vectors (Table 2). The PrP.HuAβ/PS1 (Line 85) mice are representative of all lines of mice expressing MoHuAPPswe and PS1dE9, developing cored amyloid deposits in both the cortex and hippocampus that show a wide distribution in the parenchyma (Table 2). The PrP.MoAβ/PS1 mice developed amyloid deposits in the meninges surrounding the cortex and cerebellum with additional deposits in the white-matter tracts. Since the identical vector (MoPrP.Xho) was used in generating these lines of mice, the difference in patterns of deposition cannot be easily explained by transgene expression patterns. Although we cannot rule out the possibility that the site of transgene integration modulates expression levels in some population of cells to produce these distinct patterns of deposition, the simplest explanation is that the amino acid sequence differences between human and mouse Aβ, in some manner, modulate the distribution of amyloid plaques.

**Table 2:** Characteristics of HuAβ and MoAβ mouse models

Mouse designation	PrP.HuAβ/PS1	PrP.MoAβ/PS1	PrP.HuAβ (GFP)	tet.HuAβ (107)	tet.MoAβ (GFP)	tet.HuAβ (GFP)
Aβ sequence	Human	Mouse	Human	Human	Mouse	Human
APP expression level	++	++	++	+++	++	+++
Age pathology first observed (months)	~6	~13	~12	~3	~13	~6
Cortical Pathology	P	P/M/WM	P	P	P	P
	C	C	C/D	C	D	C/D
Hippocampal Pathology	P	P/M/WM	P/M	P	P	P
	C	C	C/D	C	D	C
					(very late)	
Cerebellar Pathology	P/M/WM	P/M/WM	P/M	-	-	-
	C	C	C			

*P* Parenchymal, *M* Meningeal, *WM* White matter tract distribution, *C* Cored, *D* Diffuse morphology

The strikingly distinct pattern of deposition and morphology of deposits in the tet.MoAβ(GFP) mice is also remarkable. The distribution of the moAβ deposits shifts to the parenchyma of the cortex, and the morphology is quite distinct (Table 2). The tet.MoAβ(GFP) mice stand alone as the only one in which the amyloid deposits are primarily diffuse. However, we did observe that the PrP.HuAβ(GFP) mice, which also lack co-expressed PS1dE9, displayed a higher level of diffuse amyloid, particularly in the cortex. From these direct comparisons, it would seem that the amino acid sequence of the Aβ peptides that are deposited may have some influence on the morphology of deposited amyloid, but the co-expression of human PS1dE9 may also have some influence.

The unexpected question is: Why do the PrP.MoAβ/PS1 and tet.MoAβ(GFP) mice differ in both distribution and morphology of deposition? The two vectors systems used will be expressed in different, but overlapping populations of neurons. MoPrP.Xho is expressed in all neurons and astrocytes [4, 27] whereas the CamKII-tTA is expressed only in forebrain neurons [29]. The final processed Aβ derivatives of the APP transgenes in these mice are expected to have identical sequences. It is possible that the co-expression of PS1dE9 changes the mixture of Aβ1-40/42 and other smaller Aβ peptide derivatives, such as Aβ38, relative to the population of Aβ derivatives produced by γ-secretase containing endogenous presenilin, and that these differences in relative levels of the different derivatives underlie our observations. However, these new questions that arise from our findings do not diminish the overall conclusions of our study, which demonstrates for the first time that murine Aβ peptides possess the capacity to form amyloid deposits *in vivo*.

The degree to which murine Aβ deposits may influence cognitive behavior in mice is a topic for future study, but the late onset and distribution of amyloid in the PrP.MoAβ/PS1 mice is not indicative of a high probability of having a major impact on cognition. The tet.MoAβ(GFP) mice may offer an opportunity to examine the effects of diffusely deposited Aβ42 on cognitive function, but again the late onset of deposition creates challenges in assessing cognitive behavior.

## Conclusions

In summary, we describe, for the first time, mice that develop Alzheimer-type amyloidosis composed of murine Aβ peptides. Our findings provide a benchmark of comparison to the pathology induced in nontransgenic rodents challenged by environmental or dietary manipulations. Similar to mice expressing humanized APP genes and humans themselves, mice deposit murine Aβ in extracellular deposits that are morphologically similar to what has been described in humans (diffuse or compact). Although the age to deposit in the PP.MoAβ/PS1 mice was considerably later than that of the PrP.HuAβ/PS1 mice, despite comparable levels of expression, the first deposits in tet.MoAβ(GFP) mice were no later than that of mice expressing humanized APP transgenes at comparable levels (Table 2). Thus, we do not observe consistent differences in the rates of deposition between human and mouse Aβ. Overall, we conclude that mouse and human Aβ42 peptides have similar capacities to form amyloid *in vivo* with the sequence differences, by some manner, influencing where the amyloid deposits form and their morphological architecture.

## Additional file

Additional file 1: Table S1. Laboratory names and line designations of the transgenic mice used. **Figure S1.** Comparison of APP expression levels in different lines of APP transgenic mice. **Figure S2.** Comparison of amyloid plaque distribution in PrP.MoAβ/PS1 and PrP.HuAβ/PS1 mice. **Figure S3.** Comparison of amyloid plaque distribution in PrP.MoAβ/PS1 and PrP.HuAβ/PS1 mice. **Figure S4.** Congophilic amyloid deposits in tet.HuAβ(107), PrP.HuAβ/PS1, and PrP.MoAβ/PS1 mice. **Figure S5.** The distribution of amyloid plaques in tet.MoAβ(GFP), tet.HuAβ(107), and tet.HuAβ(GFP) mice. **Figure S6.** The distribution of amyloid plaques in 24 month old tet.MoAβ(GFP) mice. **Figure S7.** The distribution of amyloid plaques in PrP.HuAβ(GFP) mice. **Figure S8.** Analysis of insoluble Aβ in the brains of PrP.MoAβ/PS1 and PrP.HuAβ/PS1 mice. **Figure S9.** Analysis of insoluble Aβ in the brains of tet.MoAβ(GFP) mice. **Figure S10.** Neuritic morphology of amyloid plaques in the cerebellum of PrP.MoAβ/PS1 and PrP.HuAβ/PS1(Line 85) mice. (PDF 5620 kb)

## References

[1] Atwood CS, Moir RD, Huang X, Scarpa RC, Bacarra NM, Romano DM (1998). Dramatic aggregation of Alzheimer abeta by Cu(II) is induced by conditions representing physiological acidosis. J Biol Chem.

[2] Ayers JI, Xu G, Pletnikova O, Troncoso JC, Hart PJ, Borchelt DR (2014). Conformatonal specificity of the C4F6 SOD1 antibody; low frequency of reactivity in sporadic ALS cases. Acta neuropathologica communications.

[3] Borchelt DR, Ratovitski T, van Lare J, Lee MK, Gonzales V, Jenkins NA (1997). Accelerated amyloid deposition in the brains of transgenic mice coexpressing mutant presenilin 1 and amyloid precursor proteins. Neuron.

[4] Borchelt DR, Davis J, Fischer M, Lee MK, Slunt HH, Ratovitsky T (1996). A vector for expressing foreign genes in the brains and hearts of transgenic mice. Genet Anal.

[5] Borchelt DR, Thinakaran G, Eckman CB, Lee MK, Davenport F, Ratovitsky T (1996). Familial Alzheimer’s disease-linked presenilin 1 variants elevate Abeta1-42/1-40 ratio in vitro and in vivo. Neuron.

[6] Cai H, Wang Y, McCarthy D, Wen H, Borchelt DR, Price DL (2001). BACE1 is the major beta-secretase for generation of Abeta peptides by neurons. Nat Neurosci.

[7] Cheng XR, Zhou WX, Zhang YX (2014). The behavioral, pathological and therapeutic features of the senescence-accelerated mouse prone 8 strain as an Alzheimer’s disease animal model. Ageing Res Rev.

[8] Chow VW, Mattson MP, Wong PC, Gleichmann M (2010). An overview of APP processing enzymes and products. Neuromolecular Med.

[9] Crook R, Verkkoniemi A, Perez-Tur J, Mehta N, Baker M, Houlden H (1998). A variant of Alzheimer’s disease with spastic paraparesis and unusual plaques due to deletion of exon 9 of presenilin 1. Nat Med.

[10] Flood DG, Reaume AG, Dorfman KS, Lin YG, Lang DM, Trusko SP (2002). FAD mutant PS-1 gene-targeted mice: increased A beta 42 and A beta deposition without APP overproduction. Neurobiol Aging.

[11] Fraser PE, Nguyen JT, Inouye H, Surewicz WK, Selkoe DJ, Podlisny MB (1992). Fibril formation by primate, rodent, and Dutch-hemorrhagic analogues of Alzheimer amyloid beta-protein. Biochemistry.

[12] Games D, Adams D, Alessandrini R, Barbour R, Berthelette P, Blackwell C (1995). Alzheimer-type neuropathology in transgenic mice overexpressing V717F beta-amyloid precursor protein. Nature.

[13] Glenner GG, Wong CW (1984). Alzheimer’s disease and Down’s syndrome: sharing of a unique cerebrovascular amyloid fibril protein. Biochem Biophys Res Commun.

[14] Guntern R, Bouras C, Hof PR, Vallet PG (1992). An improved thioflavine S method for staining neurofibrillary tangles and senile plaques in Alzheimer’s disease. Experientia.

[15] Guo Q, Fu W, Sopher BL, Miller MW, Ware CB, Martin GM (1999). Increased vulnerability of hippocampal neurons to excitotoxic necrosis in presenilin-1 mutant knock-in mice. Nat Med.

[16] Haass C, Kaether C, Thinakaran G, Sisodia S (2012). Trafficking and proteolytic processing of APP. Cold Spring Harb Perspect Med.

[17] Hardy J, Selkoe DJ (2002). The amyloid hypothesis of Alzheimer’s disease: progress and problems on the road to therapeutics. Science.

[18] Huang X, Cuajungco MP, Atwood CS, Hartshorn MA, Tyndall JD, Hanson GR (1999). Cu(II) potentiation of alzheimer abeta neurotoxicity. Correlation with cell-free hydrogen peroxide production and metal reduction. J Biol Chem.

[19] Iwatsubo T, Odaka A, Suzuki N, Mizusawa H, Nukina N, Ihara Y (1994). Visualization of A beta 42(43) and A beta 40 in senile plaques with end-specific A beta monoclonals: evidence that an initially deposited species is A beta 42(43). Neuron.

[20] Jankowsky JL, Slunt HH, Ratovitski T, Jenkins NA, Copeland NG, Borchelt DR (2001). Co-expression of multiple transgenes in mouse CNS: a comparison of strategies. Biomol Eng.

[21] Jankowsky JL, Younkin LH, Gonzales V, Fadale DJ, Slunt HH, Lester HA (2007). Rodent A beta modulates the solubility and distribution of amyloid deposits in transgenic mice. J Biol Chem.

[22] Jankowsky JL, Melnikova T, Fadale DJ, Xu GM, Slunt HH, Gonzales V (2005). Environmental enrichment mitigates cognitive deficits in a mouse model of Alzheimer’s disease. J Neurosci.

[23] Jankowsky JL, Slunt HH, Gonzales V, Savonenko AV, Wen JC, Jenkins NA (2005). Persistent amyloidosis following suppression of Abeta production in a transgenic model of Alzheimer disease. PLoS Med.

[24] Jankowsky JL, Fadale DJ, Anderson J, Xu GM, Gonzales V, Jenkins NA (2004). Mutant presenilins specifically elevate the levels of the 42 residue beta-amyloid peptide in vivo: evidence for augmentation of a 42-specific gamma secretase. Hum Mol Genet.

[25] Kim J, Miller VM, Levites Y, West KJ, Zwizinski CW, Moore BD (2008). BRI2 (ITM2b) inhibits Abeta deposition in vivo. J Neurosci.

[26] Lee MK, Borchelt DR, Kim G, Thinakaran G, Slunt HH, Ratovitski T (1997). Hyperaccumulation of FAD-linked presenilin 1 variants in vivo. Nat Med.

[27] Lesuisse C, Xu G, Anderson J, Wong M, Jankowsky J, Holtz G (2001). Hyper-expression of human apolipoprotein E4 in astroglia and neurons does not enhance amyloid deposition in transgenic mice. Hum Mol Genet.

[28] Masters CL, Simms G, Weinman NA, Multhaup G, McDonald BL, Beyreuther K (1985). Amyloid plaque core protein in Alzheimer disease and Down syndrome. Proc Natl Acad Sci U S A.

[29] Mayford M, Bach ME, Huang YY, Wang L, Hawkins RD, Kandel ER (1996). Control of memory formation through regulated expression of a CaMKII transgene. Science.

[30] McGowan E, Pickford F, Kim J, Onstead L, Eriksen J, Yu C (2005). Abeta42 is essential for parenchymal and vascular amyloid deposition in mice. Neuron.

[31] Melnikova T, Fromholt S, Kim H, Lee D, Xu G, Price A (2012). A study of persistent and transient pathologic phenotypes in an inducible model of Alzheimer-amyloidosis and how these relate to cognitive behavior. J Neurosci.

[32] Mizushima S, Nagata S (1990). pEF-BOS, a powerful mammalian expression vector. Nucleic Acids Res.

[33] Morley JE, Kumar VB, Bernardo AE, Farr SA, Uezu K, Tumosa N (2000). Beta-amyloid precursor polypeptide in SAMP8 mice affects learning and memory. Peptides.

[34] Pardo CA, Xu Z, Borchelt DR, Price DL, Sisodia SS, Cleveland DW (1995). Superoxide dismutase is an abundant component in cell bodies, dendrites, and axons of motor neurons and in a subset of other neurons. Proc Natl Acad Sci U S A.

[35] Reeves RH, Irving NG, Moran TH, Wohn A, Kitt C, Sisodia SS (1995). A mouse model for Down syndrome exhibits learning and behaviour deficits. Nat Genet.

[36] Saito T, Matsuba Y, Mihira N, Takano J, Nilsson P, Itohara S (2014). Single App knock-in mouse models of Alzheimer’s disease. Nat Neurosci.

[37] Savonenko A, Xu H, Price DL, Borchelt DR, Markowska AL (2003). Normal cognitive behavior in two distinct lines of transgenic mice hyper-expressing mutant APPswe. Neurobiol Dis.

[38] Selkoe DJ (1989). Biochemistry of altered brain proteins in Alzheimer’s disease. Annu Rev Neurosci.

[39] Selkoe DJ, Bell DS, Podlisny MB, Price DL, Cork LC (1987). Conservation of brain amyloid proteins in aged mammals and humans with Alzheimer’s disease. Science.

[40] Semina II, Baichurina AZ, Makarova EA, Leushina AV, Kazakevich Z, Gabdrakhmanova MR (2015). Dynamics of behavioral disorders in transgenic mice with modeled Alzheimer’s disease. Bull Exp Biol Med.

[41] Steiner H, Romig H, Grim MG, Philipp U, Pesold B, Citron M (1999). The biological and pathological function of the presenilin-1 deltaexon 9 mutation is independent of its defect to undergo proteolytic processing. J Biol Chem.

[42] Tebbenkamp AT, Swing D, Tessarollo L, Borchelt DR (2011). Premature death and neurologic abnormalities in transgenic mice expressing a mutant huntingtin exon-2 fragment. Hum Mol Genet.

[43] Tebbenkamp AT, Green C, Xu G, Denovan-Wright EM, Rising AC, Fromholt SE (2011). Transgenic mice expressing caspase-6-derived N-terminal fragments of mutant huntingtin develop neurologic abnormalities with predominant cytoplasmic inclusion pathology composed largely of a smaller proteolytic derivative. Hum Mol Genet.

[44] Thinakaran G, Harris CL, Ratovitski T, Davenport F, Slunt HH, Price DL (1997). Evidence that levels of presenilins (PS1 and PS2) are coordinately regulated by competition for limiting cellular factors. J Biol Chem.

[45] Thinakaran G, Borchelt DR, Lee MK, Slunt HH, Spitzer L, Kim G (1996). Endoproteolysis of presenilin 1 and accumulation of processed derivatives in vivo. Neuron.

[46] Vallet PG, Guntern R, Hof PR, Golaz J, Delacourte A, Robakis NK (1992). A comparative study of histological and immunohistochemical methods for neurofibrillary tangles and senile plaques in Alzheimer’s disease. Acta Neuropathol.

[47] Verkkoniemi A, Kalimo H, Paetau A, Somer M, Iwatsubo T, Hardy J (2001). Variant Alzheimer disease with spastic paraparesis: neuropathological phenotype. J Neuropathol Exp Neurol.

[48] Verkkoniemi A, Somer M, Rinne JO, Myllykangas L, Crook R, Hardy J (2000). Variant Alzheimer’s disease with spastic paraparesis: clinical characterization. Neurology.

[49] Xia D, Watanabe H, Wu B, Lee SH, Li Y, Tsvetkov E (2015). Presenilin-1 knockin mice reveal loss-of-function mechanism for familial Alzheimer’s disease. Neuron.

[50] Xu G, Gonzales V, Borchelt DR (2002). Abeta deposition does not cause the aggregation of endogenous tau in transgenic mice. Alzheimer Dis Assoc Disord.

[51] Yamamoto T, Hirano A (1986). A comparative study of modified Bielschowsky, Bodian and thioflavin S stains on Alzheimer’s neurofibrillary tangles. Neuropathol Appl Neurobiol.

