# Supplementary material for: Murine Aβ over-production produces diffuse and compact Alzheimer-type amyloid deposits
Source: Acta Neuropathol Commun. 2015 Nov 14;3:72. doi: 10.1186/s40478-015-0252-9 (PMC4644287; doi:10.1186/s40478-015-0252-9)

Electronic Supplementary Material

Murine Aβ over-production produces diffuse and compact Alzheimer-type amyloid deposits

Guilian Xu[1-4], Yong Ran[1,4,] Susan E. Fromholt[1,3,4], Chunhua Fu[5], Anthony T. Yachnis[6], Todd E. Golde[1,2,4], David R. Borchelt[1-4]

[1]Center for Translational Research in Neurodegenerative Disease, [2]McKnight Brain Institute, [3]SantaFe HealthCare Alzheimer's Disease Research Center, [4]Department of Neuroscience, [5]Department of Medicine, [6]Department of Pathology, University of Florida College of Medicine, University of Florida, Gainesville, FL 32610.

Table S1. Laboratory names and line designations of the transgenic mice used.

**Mouse Designation**	**PrP.HuAβ/ PS1**	**PrP.MoAβ/ PS1**	**PrP.HuAβ (GFP)**	**tet.HuAβ (107)**	**tet.MoAβ (GFP)**	**tet.HuAβ (GFP)**
Aβ sequence	Human	**Mouse**	Human	Human	**Mouse**	Human
APP mutation	Swe	Swe	Swe/Ind	Swe/Ind	Swe/Ind	Swe/Ind
Human PS1dE9	Yes	Yes	-	-	-	-
Vector	MoPrP.Xho	MoPrP.Xho	MoPrP.Xho	CamKII-tTA X Tet-PrP	CamKII-TTA X Tet-PrP	CamKII-TTA X Tet-PrP
K14-GFP co-injection	-	-	Yes	-	Yes	Yes
**Laboratory Line Name**	**MoHuAPPswe/ PS1dE9 (Line 85)**	**MoAPPswe/ PS1dE9 (Line D943)**	**MHSI-695 +GFP**	**tet.Mo/Hu APPsi (Line 107)**	**tet.MoAPPsi +GFP**	**Tet.Mo/HuAPPsi +GFP**

Fig. S1 Comparison of APP expression levels in different lines of APP transgenic mice. The forebrains of mice from each of the lines noted on the figure were homogenized and analyzed by immunoblots with mAb 22C11 (1:1000). Examples from 2 brains of nontransgenic (NTg) mice are also shown. moAPPsi-GFP = the responder line of tet.MoAβ(GFP) mice made by co-injection with vectors for K14-GFP. tTA/moAPPsi-GFP = bigenic CamKII-tTA x tetPrP-mAPPsi (+GFP) mice. MHSI-695-GFP = mice generated by co-injection of PrP.Xho vectors encoding MoHuAPPswe/ind and K14-GFP. tTA/MoHuAPPsi (line 107) = bigenic CamKII-tTA x tetPrP-MoHuAPPsi [1]. moAPPswe/PS1dE9 (D-943) and MoHuAPPswe/PS1dE9 Line 85 are described in the text. Each lane contains 50 μg total protein analyzed on a 4-20% SDS-PAGE gel; the ages of the mice at harvest were between 1.5 to 5.5 months old. The ages that amyloid plaques usually appear in the various strains of mice are shown in the figure. The oldest observation age is 24 months for all mice.

Fig. S2 Comparison of amyloid plaque distribution in PrP.MoAβ/PS1 and PrP.HuAβ/PS1 mice.

Representative images of sagittal sections from at least 3 animals of each genotype stained by Campbell-Switzer silver stain. In these images, the background silver staining (see Figure S2) has been digitally removed to highlight the black amyloid deposits. The age of the mice that produced each image is noted on the figure.

Fig. S3 Comparison of amyloid plaque distribution in PrP.MoAβ/PS1 and PrP.HuAβ/PS1 mice.
Representative images of sagittal sections from at least 3 animals of each genotype were stained by Campbell-Switzer silver stain. Enlarged images to the right show microscopic detail for the area inside the box. Notice that the plaques in PrP.MoAβ/PS1 mice in hippocampus were primarily cored. The ages of the animals are shown.

Fig. S4 Congophilic amyloid deposits in tet.HuAβ(107), PrP.HuAβ/PS1, and PrP.MoAβ/PS1 mice.
Representative amyloid plaques and blood vessels stained by Congo red, displayed by bright field (1st column), under cross polarized light (2nd column) and auto-fluorescence (3rd and 4th columns). A-C) Cortical plaques in tet.HuAβ(107) mice (25 months old), D) amyloid deposited around cortical blood vessels of the same mouse. E-G) Cortical plaques and H) amyloid deposited in cortical blood vessels in PrP.HuAβ/PS1 mice (Line 85, 25 months old). I-K) Cerebellar plaques and L) cerebellar blood vessels in the same mouse. M-O) Cortical plaques and P) amyloid deposited in cortical blood vessels in PrP.MoAβ/PS1 mice (Line D-943, 25 months old). Q-S) Cerebellar plaques and T) cerebellar blood vessels in the same mouse.

Fig. S5 The distribution of amyloid plaques in tet.MoAβ(GFP), tet.HuAβ(107), and tet.HuAβ(GFP) mice.
Representative images of sagittal sections from at least 3 animals of each genotype stained by Campbell-Switzer silver stain. Enlarged images to the right show microscopic detail for the area inside the box. The morphology of the mouse Aβ plaques was more diffuse than either of the tet.HuAβ mice. The ages of the animals are shown.

Fig. S6 The distribution of amyloid plaques in 24 month old tet.MoAβ(GFP) mice.
Representative images of sagittal sections from at least 3 animals of each genotype stained by Campbell-Switzer silver stain. Enlarged images to the right show microscopic detail for the area inside the box. Note that all of the deposited Aβ appeared as diffuse amyloid and there was far fewer plaques in the hippocampus.

Fig. S7 The distribution of amyloid plaques in PrP.HuAβ(GFP) mice.
Representative images of sagittal sections from at least 3 animals of each genotype stained by Campbell-Switzer silver stain. Enlarged images to the right show microscopic detail for the area inside the box. The deposited Aβ in the hippocampus appeared as both cored deposits and diffuse deposits.

Fig. S8 Analysis of insoluble Aβ in the brains of PrP.MoAβ/PS1 and PrP.HuAβ/PS1 mice
A) Forebrains or cerebellum of mice at various ages were homogenized in RIPA buffer and fractionated by centrifugation to separate the insoluble Aβ, which was then re-extracted in SDS and then formic acid (FA). The levels of Aβ were measured by ELISA.

B) To demonstrate that the primary peptide detected in the insoluble fraction of the PrP.MoAβ/PS1 mice was Aβ42, we calculated the ratio of Aβ42 to 40 in the brains of each animal at each age analyzed. Data from the brains of PrP.HuAβ/PS1(Line 85) mice is shown for comparison. In the brains of the Line 85 mice, the ratio of Aβ42 to 40 in either the SDS soluble or formic acid soluble fractions ranged between 2:1 and 10:1. In the brains of the PrP.MoAβ/PS1 mice, the ratio of 42:40 ranged from 10:1 to 30:1. SDS F & SDS C = SDS soluble fractions from forebrain and cerebellum, respectively. FA F & FA C = Formic acid soluble fractions from forebrain and cerebellum, respectively.

Fig. S9 Analysis of insoluble Aβ in the brains of tet.MoAβ(GFP) mice
Forebrains of mice were homogenized in RIPA buffer and then fractionated by centrifugation and sequential extraction in SDS and formic acid (FA) to separate the soluble and insoluble Aβ peptides. As the mice aged and amyloid was deposited, there was a significant rise in the level of Aβ42 that was solubilized by SDS. Consequently, the ratio of Aβ42 to 40 in the SDS-soluble fraction rose to very high levels.

Fig. S10 Neuritic morphology of amyloid plaques in the cerebellum of PrP.MoAβ/PS1 and PrP.HuAβ/PS1(Line 85) mice
Representative images of neuritic profiles in cerebellar, hippocampal, and cortical amyloid plaques in tissue sections stained with antibodies to ubiquitin. The images shown are representative of images from an analysis of at least 3 animals at each age and each genotype with at least 6 individual tissue sections taken from similar levels for each animal. A-F). PrP.HuAβ/PS1(Lline 85); G-L). PrP.MoAβ/PS1 mice. All the images were digitally cropped from the pictures taken with 100x oil lens; further digital magnification was used to enlarge the image (all images digitally enlarged to the same degree).

Fig. S1. Expression of total APP in transgenic mice.

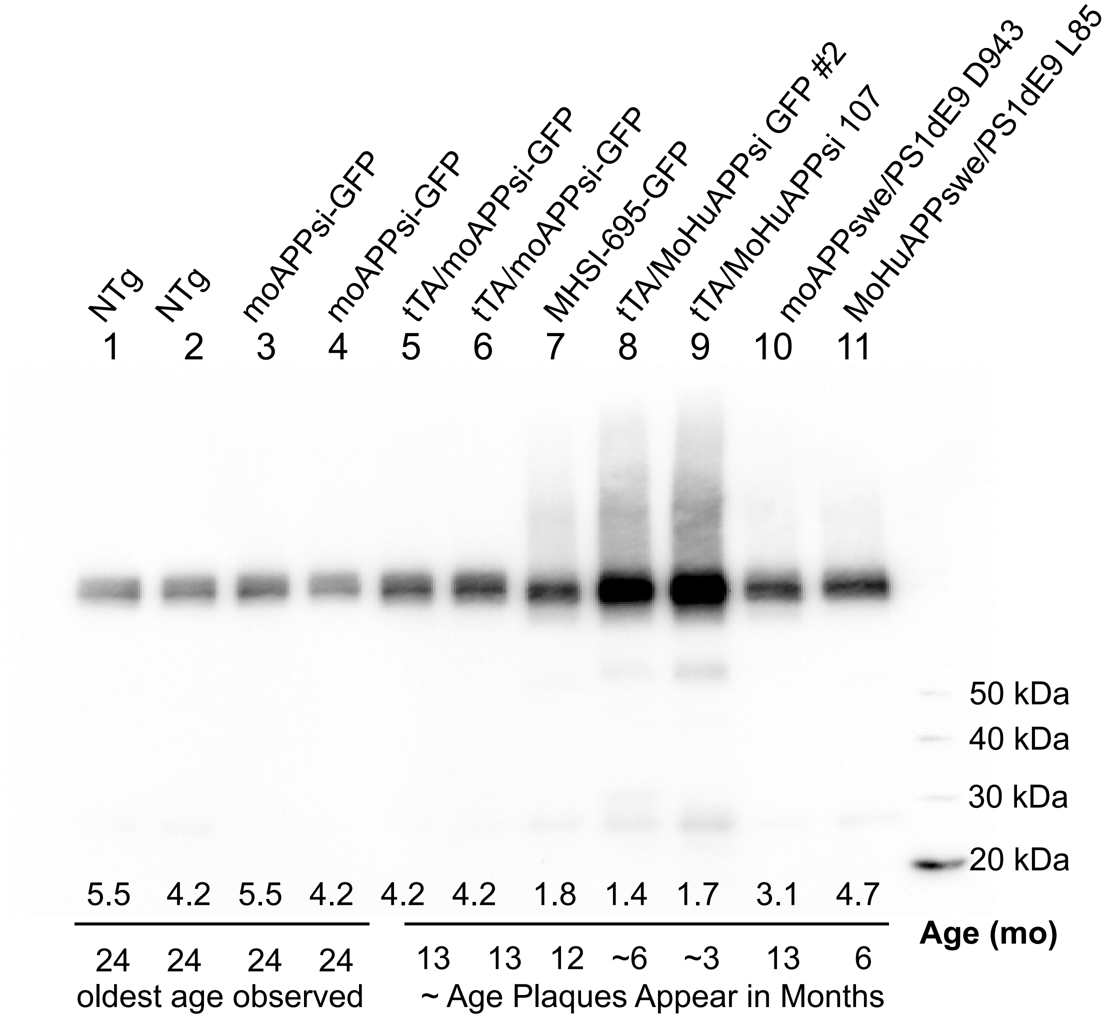

Fig. S2. Distribution of amyloid deposition in PrP.MoAβ/PS1 and PrP.HuAβ/PS1 mice.

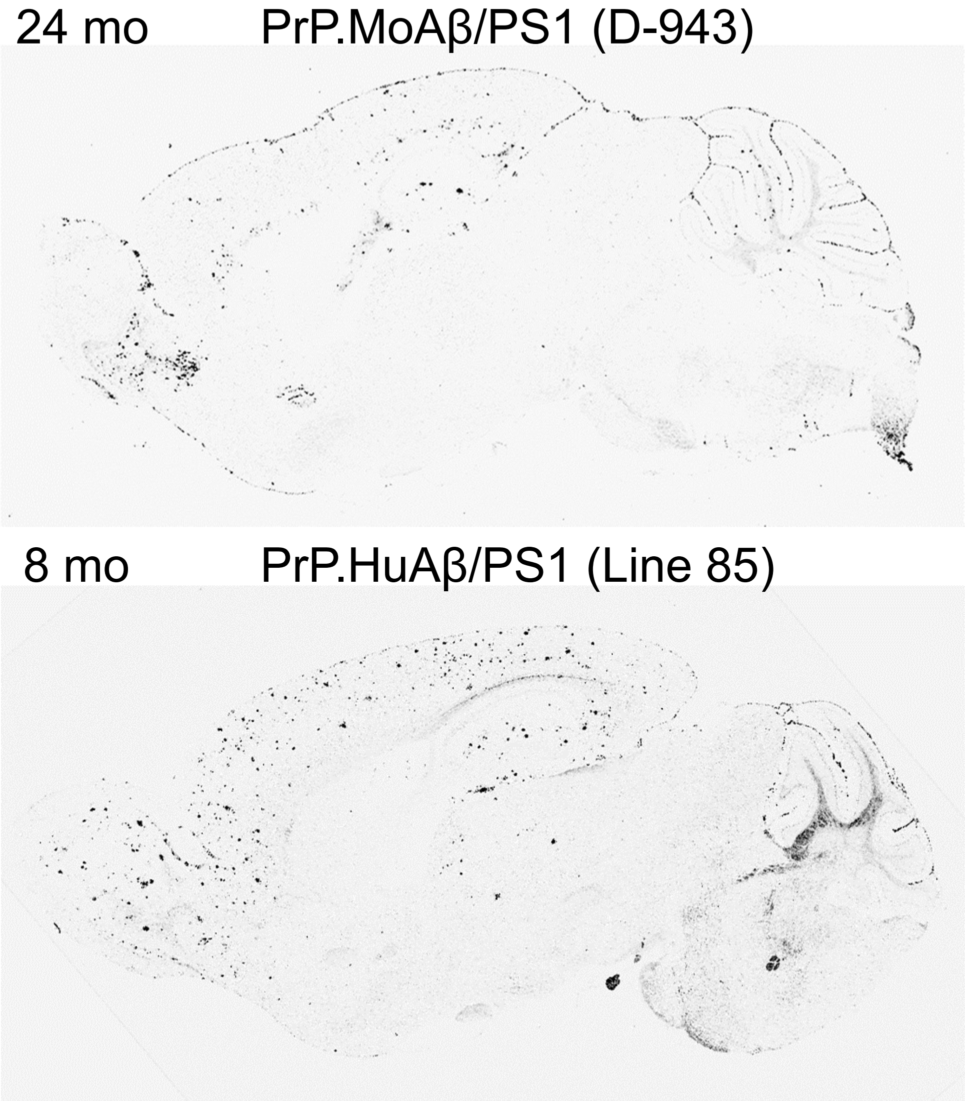

Fig. S3. The distribution of amyloid plaques in PrP.MoAβ/PS1 and PrP.HuAβ/PS1 mice.

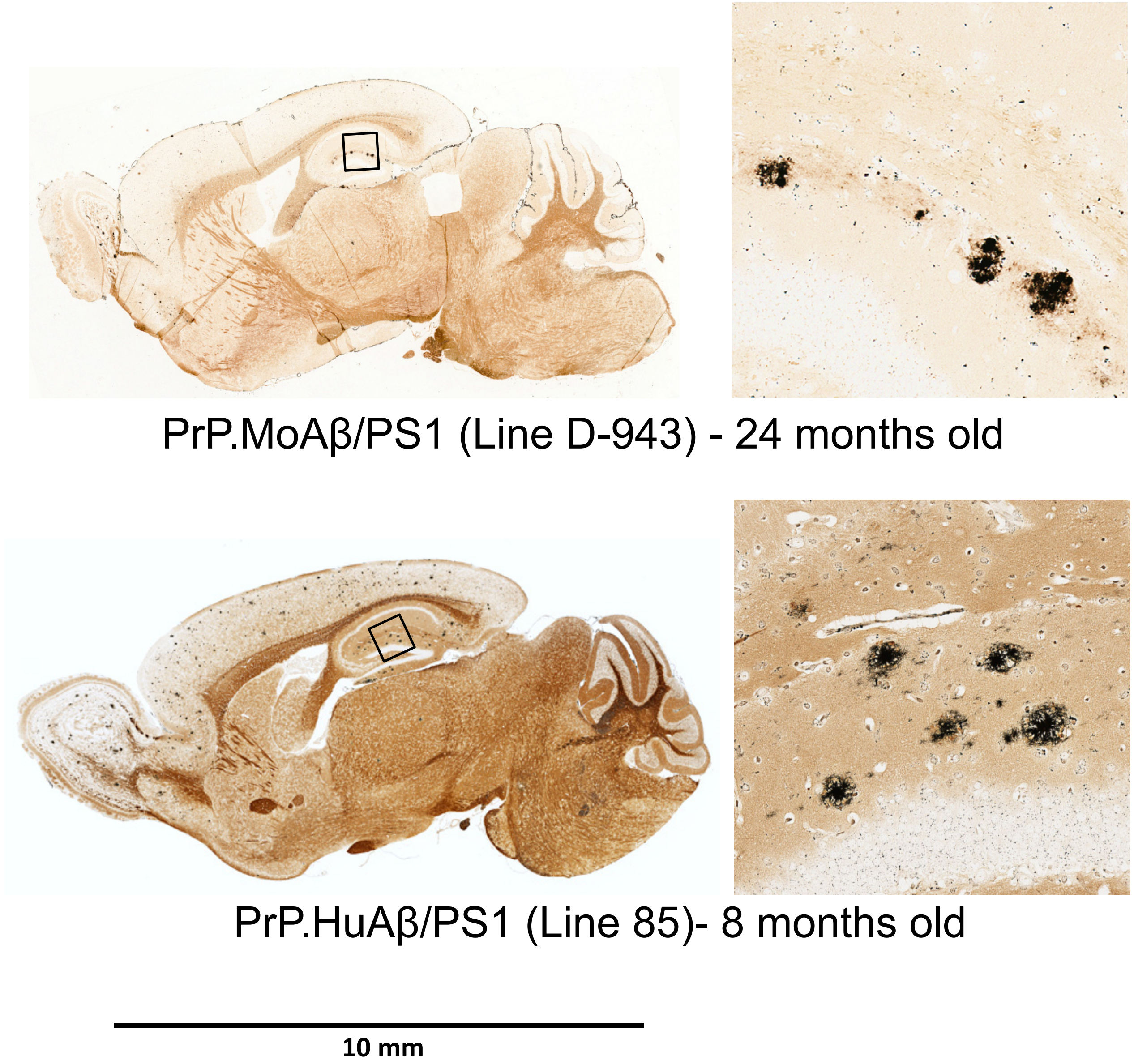

Fig. S4. Congo Red staining of cored amyloid deposits.

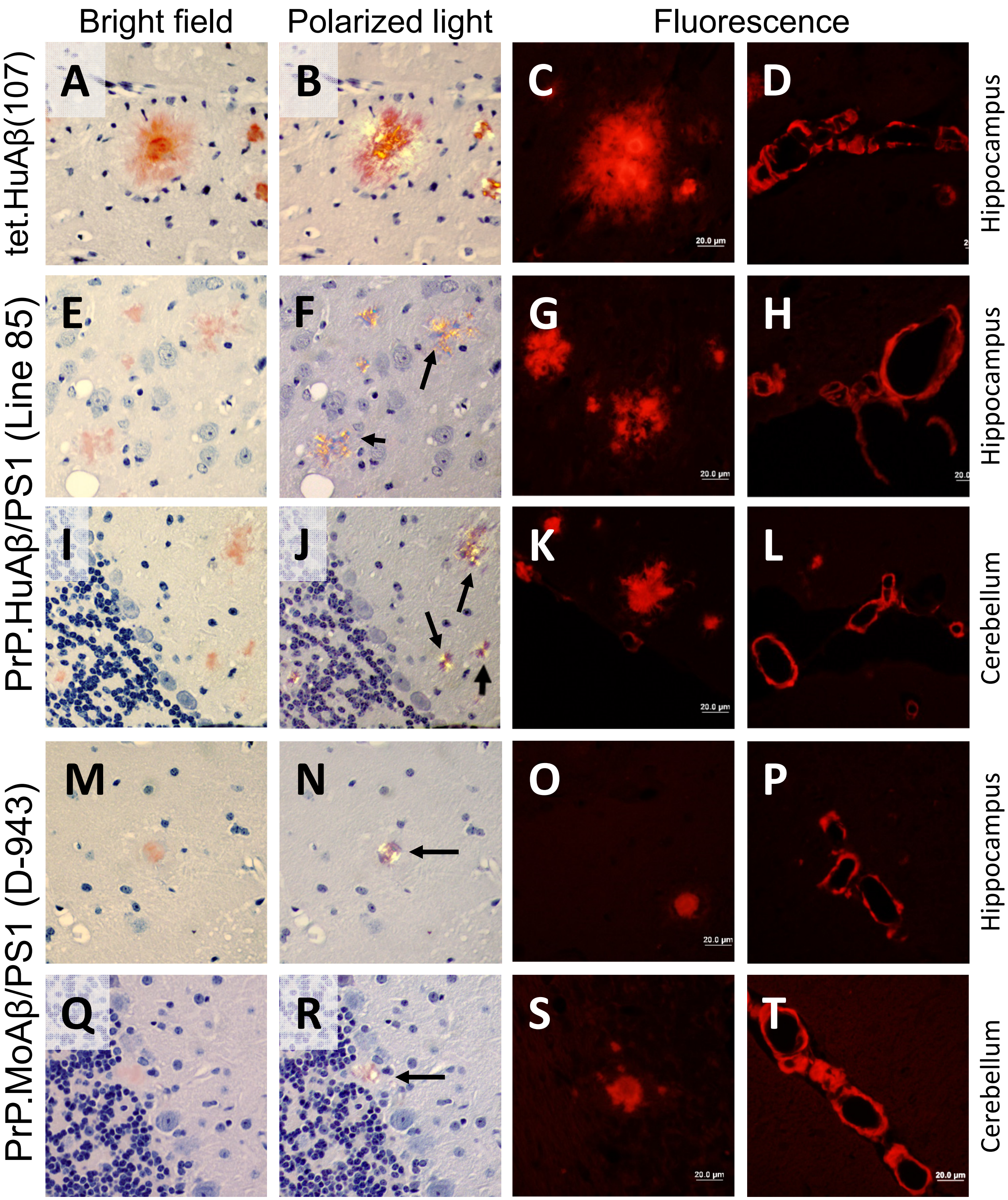

Fig. S5. The distribution of amyloid plaques in tet.MoAβ(GFP), tet.HuAβ(107), and tet.HuAβ(GFP) mice.

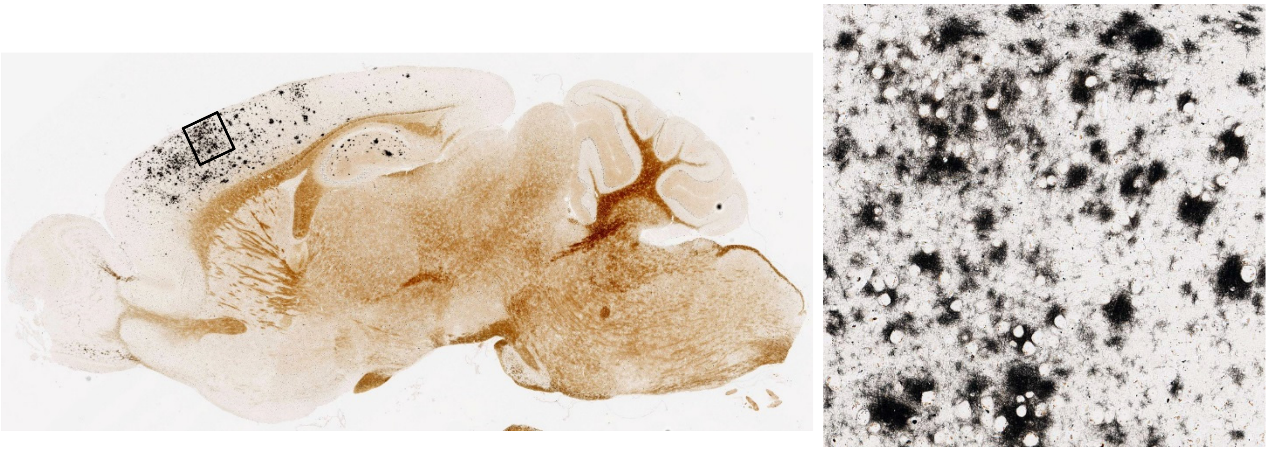

tet.MoAβ(GFP)- 18 months old

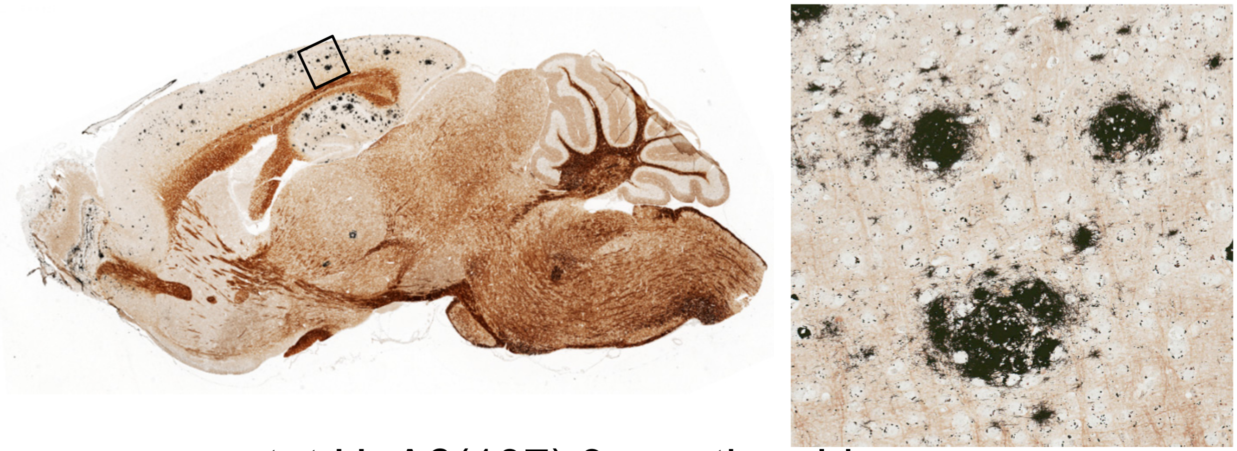

tet.HuAβ(107) 9 months old

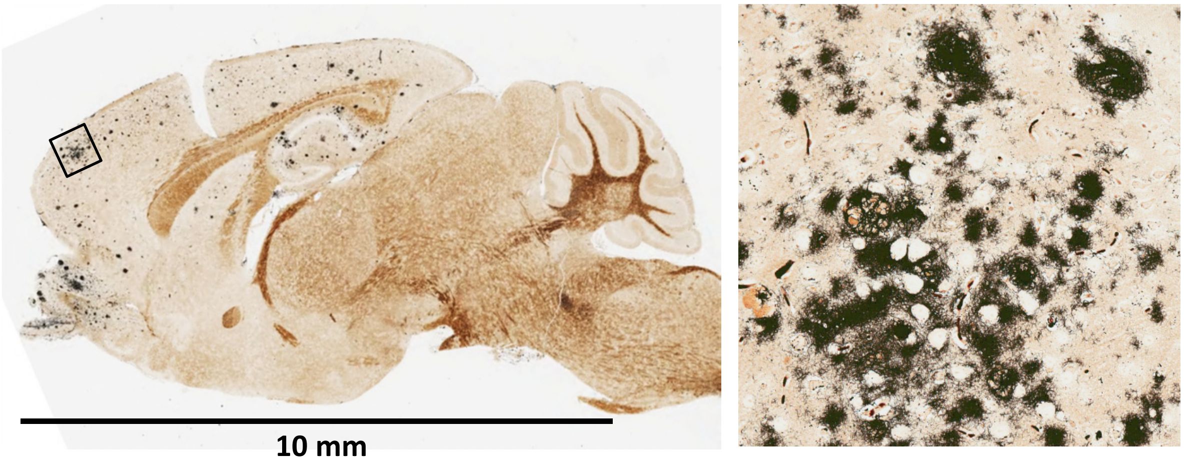

tet.HuAβ(GFP) - 7 months old

Fig. S6 . The distribution of amyloid plaques in very old tetMoAβ(GFP) mice.

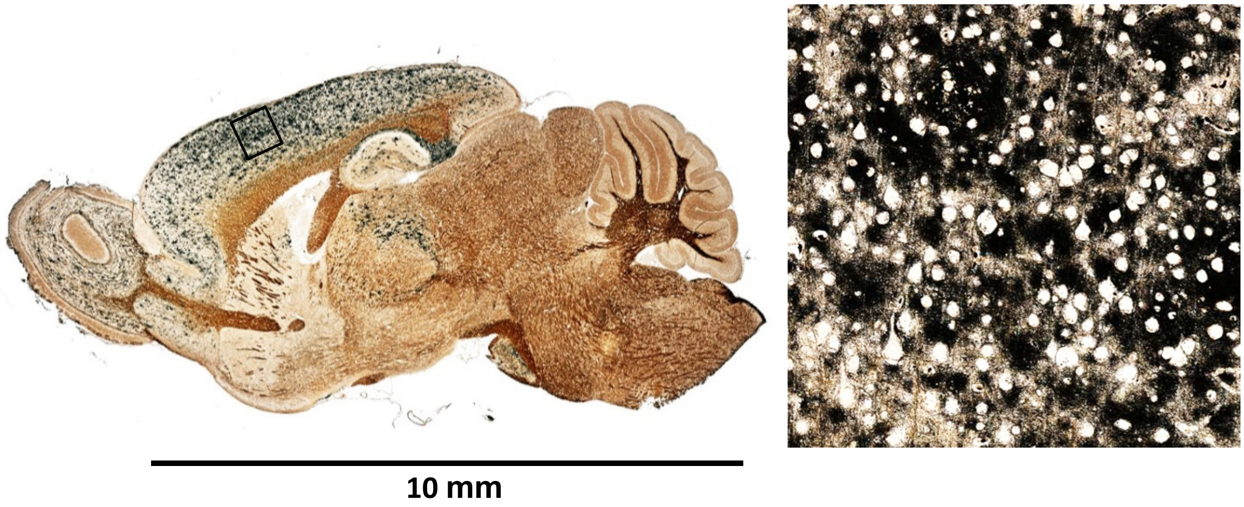

tet.MoAβ(GFP) - 24 months old

Fig. S7. The distribution of amyloid plaques in PrP.HuAβ(GFP) mice.

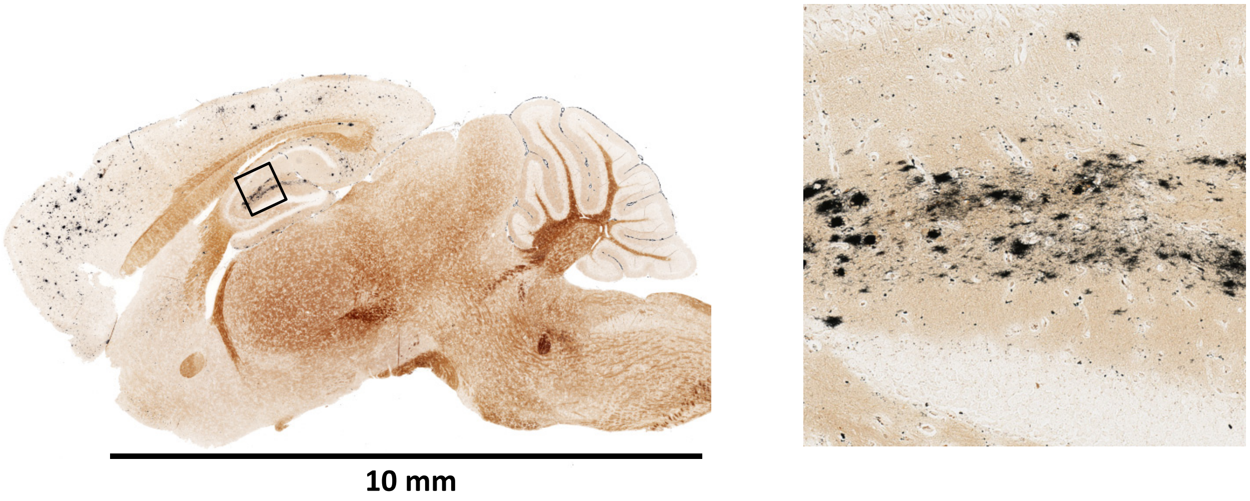

PrP.HuAβ(GFP) - 20 months old

Fig. S8A. Comparisons of the levels of Aβ in SDS- and FA-soluble fractions analyzed by ELISA between transgenic mice expression mouse or human Aβ.

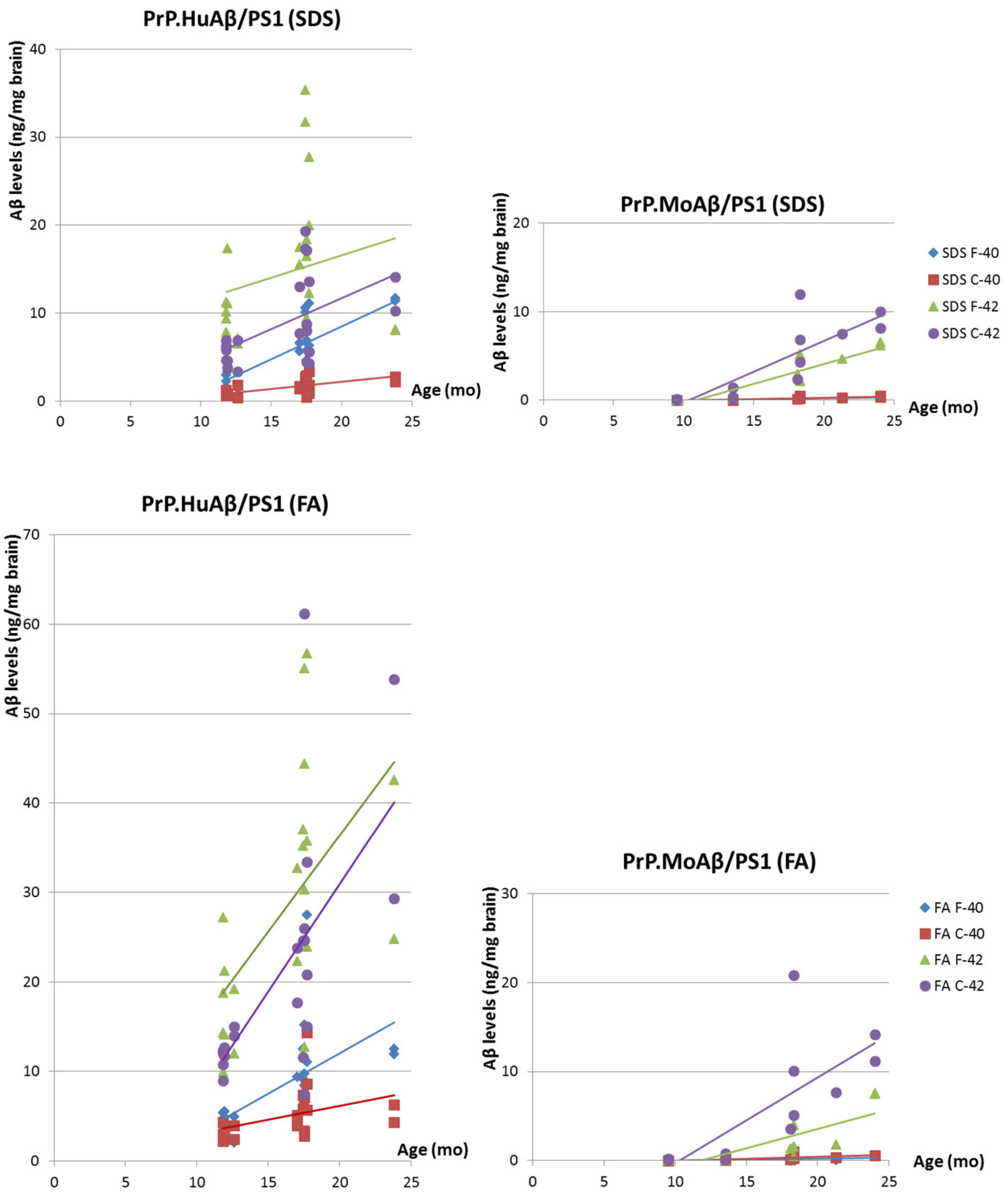

Fig. S8B. Comparison of the ratios of Aβ 42/40 in SDS- and FA-soluble fractions analyzed by ELISA between transgenic mice expression mouse or human Aβ.

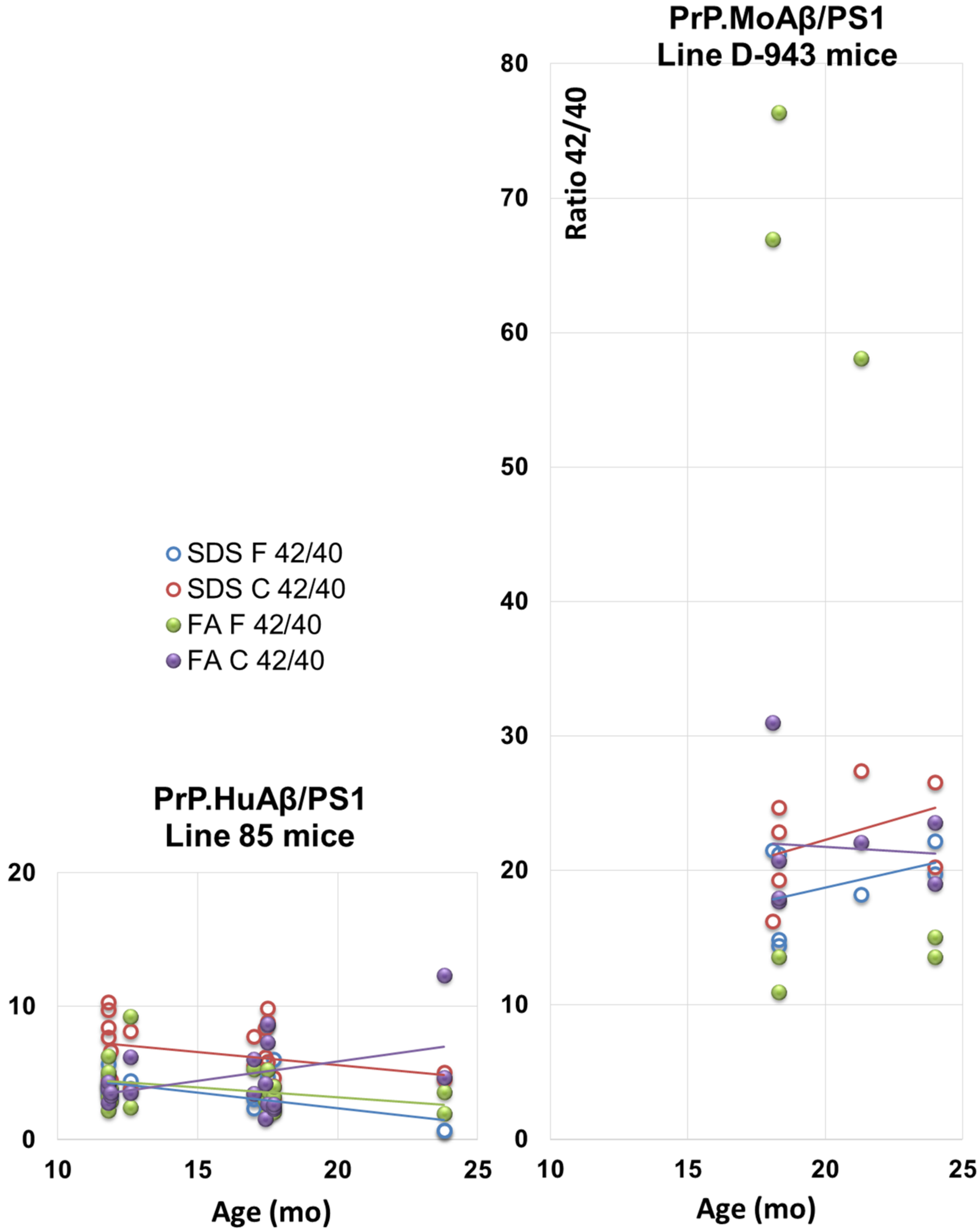

Fig. S9. Analysis of SDS- and FA- soluble fractions of mouse Aβ by ELISA in tet.MoAβ(GFP) mice.

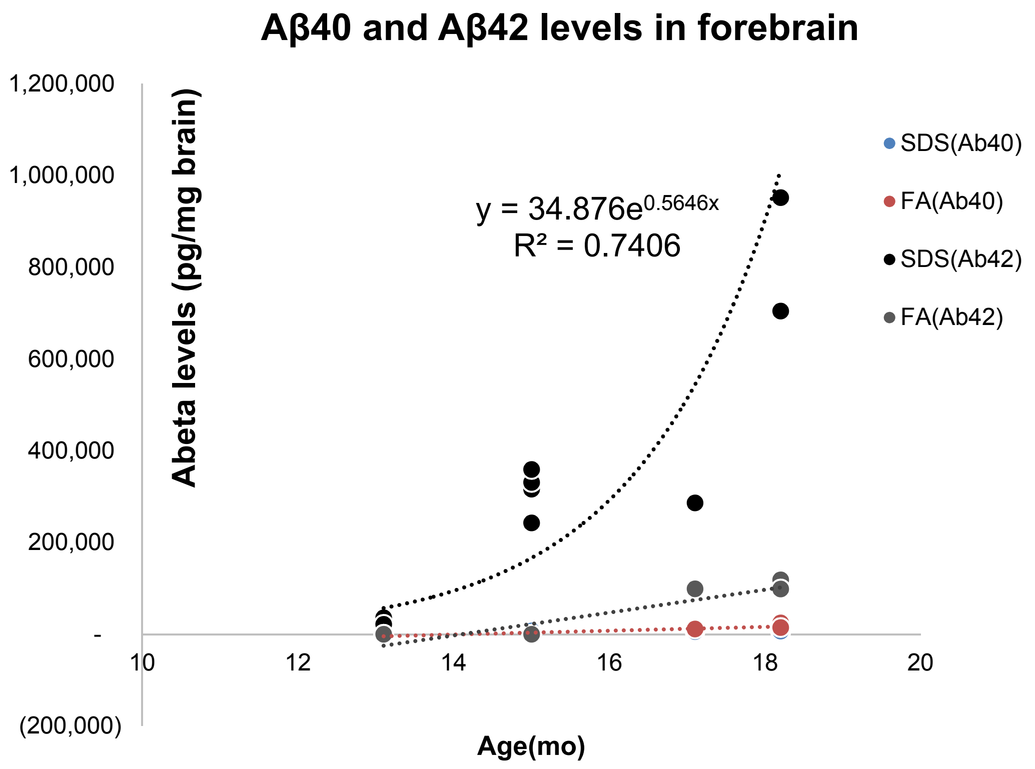

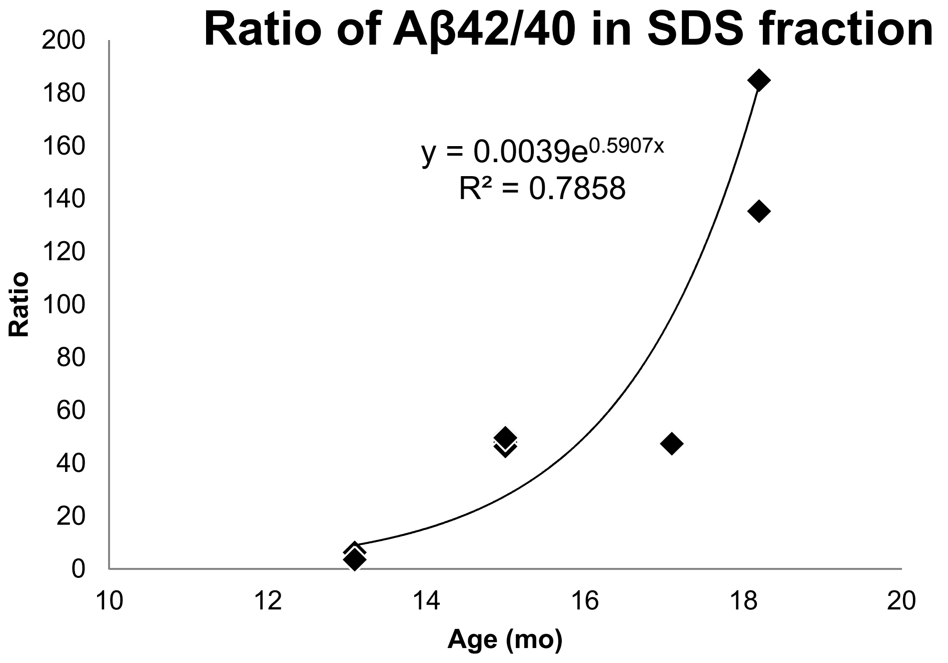

Fig. S10. Neuritic morphology of amyloid plaques in the cerebellum of PrP.HuAβ/PS1 (Line 85) and PrP.MoAβ/PS1 (D-943) mice.

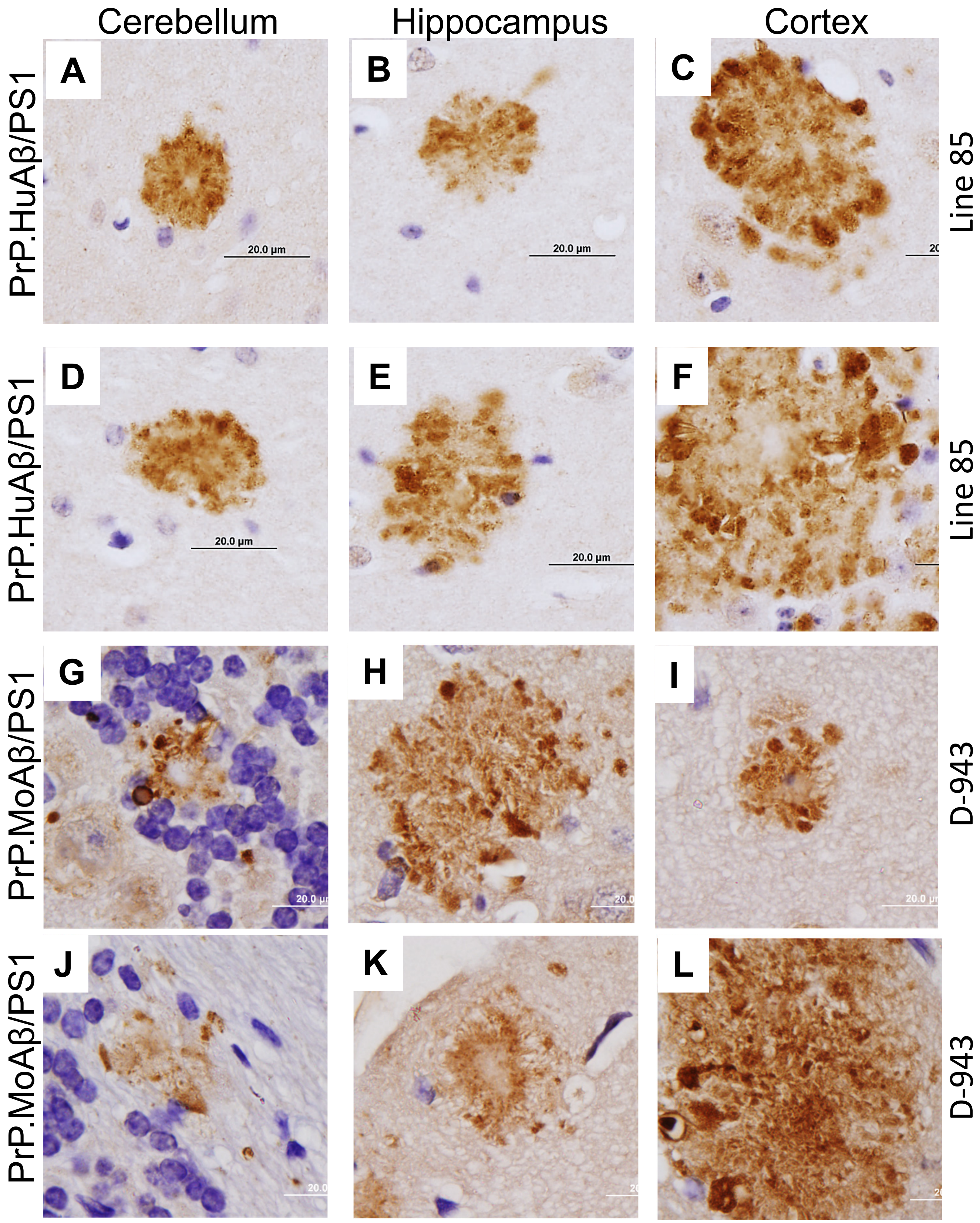

Supplement: Additional file 1: Table S1. — Laboratory names and line designations of the transgenic mice used. Figure S1. Comparison of APP expression levels in different lines of APP transgenic mice. Figure S2. Comparison of amyloid plaque distribution in PrP.MoAβ/PS1 and PrP.HuAβ/PS1 mice. Figure S3. Comparison of amyloid plaque distribution in PrP.MoAβ/PS1 and PrP.HuAβ/PS1 mice. Figure S4. Congophilic amyloid deposits in tet.HuAβ(107), PrP.HuAβ/PS1, and PrP.MoAβ/PS1 mice. Figure S5. The distribution of amyloid plaques in tet.MoAβ(GFP), tet.HuAβ(107), and tet.HuAβ(GFP) mice. Figure S6. The distribution of amyloid plaques in 24 month old tet.MoAβ(GFP) mice. Figure S7. The distribution of amyloid plaques in PrP.HuAβ(GFP) mice. Figure S8. Analysis of insoluble Aβ in the brains of PrP.MoAβ/PS1 and PrP.HuAβ/PS1 mice. Figure S9. Analysis of insoluble Aβ in the brains of tet.MoAβ(GFP) mice. Figure S10. Neuritic morphology of amyloid plaques in the cerebellum of PrP.MoAβ/PS1 and PrP.HuAβ/PS1(Line 85) mice. (PDF 5620 kb) [file 40478_2015_252_MOESM1_ESM.pdf]
